# Serotypes, Virulence-Associated Factors, and Antimicrobial Resistance of *Streptococcus suis* Isolates Recovered From Sick and Healthy Pigs Determined by Whole-Genome Sequencing

**DOI:** 10.3389/fvets.2021.742345

**Published:** 2021-11-02

**Authors:** Maverick Aradanas, Zvonimir Poljak, Nahuel Fittipaldi, Nicole Ricker, Abdolvahab Farzan

**Affiliations:** ^1^Pathobiology, Ontario Veterinary College, University of Guelph, Guelph, ON, Canada; ^2^Population Medicine, Ontario Veterinary College, University of Guelph, Guelph, ON, Canada; ^3^Faculty of Veterinary Medicine, University of Montreal, Saint-Hyacinthe, QC, Canada

**Keywords:** *Streptococcus suis*, whole genome sequencing, *in silico* serotyping, virulence factors, antimicrobial resistance, swine

## Abstract

*Streptococcus suis* is ubiquitous in swine, and yet, only a small percentage of pigs become clinically ill. The objective of this study was to describe the distribution of serotypes, virulence-associated factor (VAF), and antimicrobial resistance (AMR) genes in *S. suis* isolates recovered from systemic (blood, meninges, spleen, and lymph node) and non-systemic (tonsil, nasal cavities, ileum, and rectum) sites of sick and healthy pigs using whole-genome sequencing. In total, 273 *S. suis* isolates recovered from 112 pigs (47 isolates from systemic and 136 from non-systemic sites of 65 sick pigs; 90 isolates from non-systemic sites of 47 healthy pigs) on 17 Ontario farms were subjected to whole-genome sequencing. Using *in silico* typing, 21 serotypes were identified with serotypes 9 (13.9%) and 2 (8.4%) as the most frequent serotypes, whereas 53 (19.4%) isolates remained untypable. The relative frequency of VAF genes in isolates from systemic (Kruskal–Wallis, *p* < 0.001) and non-systemic (Kruskal–Wallis, *p* < 0.001) sites in sick pigs was higher compared with isolates from non-systemic sites in healthy pigs. Although many VAF genes were abundant in all isolates, three genes, including *dltA* [Fisher's test (FT), *p* < 0.001], *luxS* (FT, *p* = 0.01), and *troA* (FT, *p* = 0.02), were more prevalent in isolates recovered from systemic sites compared with non-systemic sites of pigs. Among the isolates, 98% had at least one AMR gene, and 79% had genes associated with at least four drug classes. The most frequently detected AMR genes were *tetO* conferring resistance to tetracycline and *ermB* conferring resistance to macrolide, lincosamide, and streptogramin. The wide distribution of VAFs genes in *S. suis* isolates in this study suggests that other host and environmental factors may contribute to *S. suis* disease development.

## Introduction

*Streptococcus suis* is a Gram-positive opportunistic bacterial pathogen responsible for meningitis and other diseases in pigs and occasionally capable of zoonotic infections (1). *S. suis* is ubiquitous in pigs, and although only a small percentage of pigs become clinically ill (2–4), the disease can cause significant economic losses to swine producers.

The routine use of antimicrobials to control *S. suis* infection also presents a serious problem in the development of antimicrobial resistance (AMR) (5). AMR in *S. suis* has been documented worldwide with high rates of phenotypic resistance to tetracyclines, lincosamides, and macrolides reported (6–11). Although in lower frequencies, resistance to other antimicrobials including beta-lactams (12–14), trimethoprim–sulfamethoxazole (11, 13, 15), aminoglycosides (6, 10, 11), fluoroquinolones (10, 16–18), and chloramphenicol (19) has also been reported. Over time, there has been a global trend of increased resistance to tetracycline, aminoglycosides, fluoroquinolones, macrolides, and cephalosporins (8, 20, 21) in *S. suis*. Recent metagenomic studies have also supported the trend of increased resistance and broad distribution of AMR-associated genes globally (20, 22, 23). Recombination plays a key role in the evolutionary history of *S. suis* (24), and it has been suggested that *S. suis* has the potential to act as an AMR reservoir (8).

Currently, 29 *S. suis* serotypes are recognized, with serotypes 1, 1/2, 2, 3, 5, 7, and 14 being the most frequent serotypes associated with clinical infections in North America (20). Globally, the key serotypes associated with clinical infections consist primarily of serotypes 1/2, 2, and 3 (20), with serotype 2 being the most prevalent and well-characterized in human and pig clinical infections. Recently, serotype 9 has shown increased importance in *S. suis* clinical pig infections globally with increased prevalence in many European countries, including Spain, Netherlands, Germany, and Belgium (5, 20, 25), as well as in China (26) and Canada (27, 28).

Serotyping of *S. suis* is traditionally conducted by slide co-agglutination test using antibodies directed against the capsular polysaccharide (CPS) (29). However, over the last two decades, multiplex polymerase chain reaction (PCR) assays based on conserved genes and *cps* gene clusters have also been developed as alternatives to serotyping *S. suis* (30–32). Co-agglutination and PCR methods have been unable to distinguish certain serotypes from each other, namely between serotypes 1 and 14 and serotypes 2 and 1/2. Furthermore, both methods often result in a proportion of isolates being untypables, i.e., unable to be assigned to any of the currently known serotypes. The serotypes that previously could not be distinguished (ex. 1 and 14; 2 and 1/2) can now be identified based on single-nucleotide polymorphisms (SNPs) in the *cps* genes either by mismatch amplification mutation assay (33) or through *in silico* methods using whole-genome sequencing (WGS) data (34).

Multilocus sequence typing (MLST) is also commonly used for subtyping *S. suis* strains. The MLST for *S. suis* was first established by King et al. (35), who developed the model that assigns a sequence type (ST) based on the allele combinations of seven housekeeping genes (*recA, aroA, thrA, cpn60, mutS, dpr*, and *gki*). *The* serotype 2 strains have been extensively characterized by MLST, and ST1 has been shown to be the most frequently associated with disease in both humans and pigs in Europe, Asia, Africa, and South America (20, 35, 36). Conversely, in North America, ST25 has a high prevalence and association with disease in pigs and humans (20, 37, 38).

The exact virulence mechanisms of *S. suis* are not well-understood. Numerous virulence-associated factors (VAFs) have been suggested to be involved in *S. suis* pathogenesis. The most studied VAFs include the CPS (39, 40), muramidase-released protein (MRP) (41, 42), extracellular protein factor (EPF) (43), and suilysin (SLY) (44). However, the actual involvement of many VAFs in *S. suis* disease development remains controversial (45). Three key VAFs have been characterized as important for virulence in serotype 2 isolates (EPF, SLY, and MRP); however, virulent *S. suis* strains that do not produce or carry genes encoding these proteins are regularly reported in the literature (45). Furthermore, several VAFs, including LysM (46), EsxA (47), SrtR (48), 5′-nucleotidase (*ssadS*) (49), and antigen I/II (50), have been found to be associated with clinical serotype 9 strains but not serotype 2 strains.

The objectives of this study were to analyze the WGS data of *S. suis* isolates recovered from systemic and non-systemic sites of sick and healthy pigs from multiple farms to determine the serotype of the *S. suis* isolates using an *in silico* method and identify the distribution of VAFs and AMR genes across the different serotypes, farms, and disease status in those isolates.

## Materials and Methods

### *Streptococcus suis* Isolates

The isolates used in this study were recovered from nursery pigs with clinical signs of *S. suis* infection and from age-matched healthy animals on the same farm whenever possible on Ontario farms between 2013 and 2018 (27, 51). The identities of *S. suis* isolates were considered confirmed if glutamate dehydrogenase (*gdh*) (32) and recombination protein N (*recN*) (52) were both detected by PCR (27, 51). A total of 294 *S. suis* isolates were subjected to WGS, but 21 isolates were excluded due to contig N50 values < 10,000 and/or contamination in the final assembly. The isolates were recovered from systemic (blood, meninges, spleen, and lymph node) and non-systemic (tonsil, nasal, ileum, and rectal) sites of 112 pigs on 17 farms: 183 isolates from 65 sick pigs and 90 isolates from 47 healthy pigs. Sick pigs displayed one or more *S. suis* infection clinical signs such as ataxia, paralysis, shaking, paddling, opisthotonos, convulsions, nystagmus, and/or incoordination.

The isolates were grouped into four different categories: Systemic—confirmed (SC), non-systemic—confirmed (NSC), non-systemic—probable (NSP), and non-systemic—healthy (NSH) (Table 1). The SC (*n* = 47) and NSC (*n* = 65) isolates were recovered from systemic and non-systemic sites of 32 pigs, respectively, that were both symptomatic and confirmed to have *S. suis* recovered from at least one systemic site. The NSP (*n* = 71) isolates were obtained from non-systemic sites of 33 symptomatic pigs, for which *S. suis* was not recovered from any systemic site. The NSH isolates (*n* = 90) were recovered from 47 healthy pigs.

**Table 1:** Distribution of 273 isolates recovered from sick and healthy pigs into isolate groups.

**Isolate group**	**Number (%) of isolates**	**Isolation sites**	**Clinical signs of *S. suis* infection**
Systemic—confirmed (SC)	47 (17)	Blood, meninges, spleen, lymph node	Yes
Non-systemic—confirmed (NSC)	65 (24)	Tonsil, nasal cavities, ileum, rectum	Yes
Non-systemic—probable (NSP)	71 (26)	Tonsil, nasal cavities, ileum, rectum	Yes
Non-systemic—healthy (NSH)	90 (33)	Tonsil, nasal cavities, rectum	No

### Whole-Genome Sequencing

The *S. suis* isolates were plated on phenylethyl alcohol agar and incubated at 35°C with 5% CO_2_ for 48 h. Then, DNA was extracted using Qiagen DNeasy Blood and Tissue kit (Qiagen, Hilden, Germany) according to the protocol provided by the manufacturer. WGS of 294 isolates was done by HiSeqX PE150 (242 isolates) or NovaSeq6000 (36 isolates) by Genome Quebec (Montreal, QC, Canada), and 16 isolates were sequenced using MiSeq by the Laboratory Services at the University of Guelph (ON, Canada).

### *In silico* Serotyping

The *S. suis* sequences were typed through the pipeline SsuisSerotyping_pipeline (34) (https://github.com/streplab/SsuisSerotyping_pipeline). Conflicting serotype assignments for an isolate between PCR and *in silico* serotyping outputs were addressed by comparisons of the *cps* genes as described by Athey et al. (34) (Supplementary Figure 1), and the synteny of the *cps* loci of isolates to reference loci was visualized using easyFig (53).

### Genome Assembly

WGS raw reads for each isolate were trimmed with Trimmomatic 0.39 (54) using recommended parameters for Illumina paired-end reads with a minimum sequence length of 100 bp. The trimmed reads were checked for sequence quality with FastQC 0.11.8 (55). The trimmed sequences were then assembled *de novo* with the genome assembly program SPAdes 3.13.1 (56) with the “—careful” flag. Assemblies with contig N50 values lower than 10,000 were excluded (*N* = 21, from 294 total isolates to 273 final isolates). The quality of the assemblies was assessed using BUSCO (57) with the Lactobacillales database to check for missing data, duplication, and fragmentation. Furthermore, potential contaminants were assessed by determining the identities of contig assemblies against a bacterial genome database with Kraken2 (58) with default parameters.

### Multilocus Sequence Typing

MLST was determined using SRST2 0.2.0 (59) using the trimmed read sets for each isolate. The *S. suis* MLST database used was from PubMLST (https://pubmlst.org/) accessed in December 2020.

### Annotation and Gene Presence/Absence

The assembled genomes were annotated with Prokka 1.14.6 (60). Annotation with Prokka used a curated local reference database of published *S. suis* strains (Supplementary Table 1). The *S. suis* P1/7 serotype 2 strain annotation was set as the highest priority reference genome. The gene product labels for VAFs within the reference sequence file input for Prokka were manually annotated to confirm consistency with published gene products (61). Annotated contigs were used as inputs into ROARY 3.13.0 (62) to determine gene presence, which was used further for downstream analyses.

### Virulence-Associated Factors

The VAFs of interest in this study were putative proteins involved in *S. suis* pathogenesis reviewed by Fittipaldi et al. (40). Additionally, five VAFs found to play important roles in the virulence of *S. suis* serotype 9 strains were included in the analysis: LysM (46), EsxA (47), SrtR (48), 5′-nucleotidase (*ssadS*) (49), and antigen I/II (50).

The isolates were also classified into eight different unique genotypes based on the presence of three classical virulence genes (*mrp, sly, epf*, and epf^*^) mainly associated with serotype 2 (63). The gene epf^*^ is a larger variant of *epf* that was reported to be less virulent in pigs (64).

### Confirmation of Gene Presence

TBLASTN from the BLAST+ 2.10.1 suite (65) was used to compare VAF gene protein sequences with all the assembled genomes to confirm the presence of VAF genes. A gene was considered present if a positive hit was identified with >80% percent protein identity and >80% sequence coverage. Further confirmations of individual genes were performed by mapping of reads using bowtie2 2.4.1 (66) and samtools 1.11 (67) on VAF genes of interest.

### Antimicrobial Resistance

CARD's Resistance Gene Identifier (RGI, 5.1.1) (68) software was used to determine the presence of AMR genes using CARD database version 3.1.0. The analysis used three models supported by RGI, including CARD's protein homolog models, protein variant models, and ribosomal RNA mutation models. The cut-off was set to include only perfect and strict paradigms. SNP screening was not included in the analysis.

### Statistical Analysis

RStudio 1.2.1335 and R 3.6.1 were used for downstream analysis, visualization, and statistical analysis. McNemar's test, using the function “mcnemar.test” from the base R package, was used to determine if there was a significant difference between the assigned serotypes by PCR and *in silico*. To compare the overall distribution patterns of VAFs between and within isolate groups (SC, NSC, NSP, and NSH), relative frequency (RF) for each gene was calculated at the group level as previously described by Weinert et al. (24). RF was defined as $RF=\;\frac{F_{group}}{F_{total}}$, where *F*_*group*_ is the proportion of isolates carrying the gene within an isolate group and the *F*_*total*_ is the proportion of isolates carrying the gene out of the isolates from all four groups. An RF > 1 would indicate a higher frequency of the gene in the group compared with its overall frequency within the data set (all groups) and is interpreted as gene overrepresentation within that group. Kruskal–Wallis rank-sum test of mean relative frequencies, using the R base function kruskal.test(), was used to compare the mean RF of VAF genes among the isolates in two different ways—(i) VAF RF between isolate groups and (ii) VAF RF compared with the RF of all non-VAF genes carried by isolates within each isolate group. The same approach was also taken in testing differences in the distribution of AMR genes between the four isolate groups.

Fisher's Exact Test for Count Data, using the R function fisher.test(), was used to test whether there was a significant difference between proportions of isolates that carried prevalent VAF genes between isolate groups. If a gene was found to have a significantly higher frequency in SC isolates, the potential effects of farm and serotype in its frequency were investigated through mixed-effect logistic regression using the “lme” function from R package “lme4” and restricted maximum likelihood method. The presence and absence of the gene were the outcomes of the model with isolate groups (SC, NSC, NSP, and NSH) as predictor variables and farm source and serotypes as random variables. The NSH group was set as the reference for the isolate groups. The *p*-values were adjusted using the base R function “p. adjust” with the method set as “BH” (Benjamini–Hochberg procedure). The significant effects of each random effect variable to the regression model were determined using the “rand” function from the R package “lmerTest.”

Non-metric multidimensional scaling (NMDS) analysis was used to determine if the isolates from specific serotypes are clustered based on their VAF gene profiles. Only serotypes with a frequency >5% (2, 3, 9, 16, 29, and untypable isolates) were included, whereas other serotypes below 5% frequency were grouped under the category “others.” The NMDS was performed using the function metaMDS from the “vegan” package. The distance matrix used for the NMDS was calculated using the Jaccard similarity index based on the presence and absence of VAF genes in each isolate.

An association plot, using the function “assoc” from R package “vcd,” was used to visualize a contingency table of the presence of AMR genes within serotypes. The formula parameter was left at default to using Pearson's chi-squared test of independence. AMR genes found in less than five isolates were excluded. The plot highlighted cases when the observed number of an AMR gene within a serotype differed from the expected values (either lower or higher), under the assumption that the presence of the AMR gene was independent of serotype. Higher observed number indicated a potential association of an AMR gene with a serotype(s) within the entire data set. To determine if the association of a gene to a serotype is valid, a chi-squared test was performed on a separate AMR gene presence and absence contingency table for said serotype.

## Results

### *In silico* Serotyping

**Figure 1:**
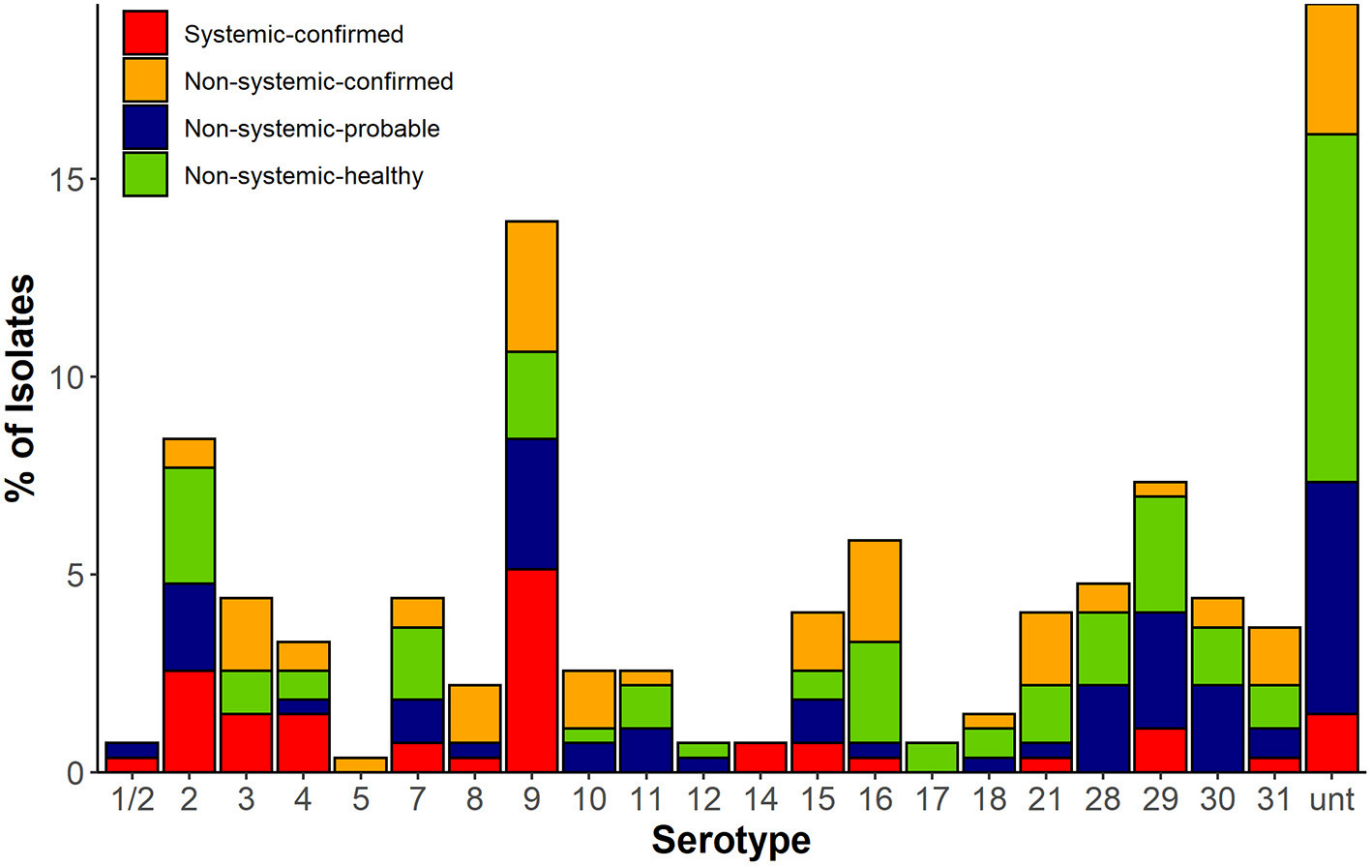
*In silico* serotype of 273 *S. suis* isolates in four classified groups based on disease status (confirmed, probable infection, and healthy) and isolation source. Systemic sites included blood, meninges, spleen, and lymph node. Non-systemic sites included tonsil, nasal cavities, ileum, and rectal sample.

Twenty-one serotypes were identified using *in silico* typing (Figure 1). The most common serotypes recovered were serotype 9 (38/273, 14%), serotype 2 (23/273, 8%), serotype 16 (16/273, 6%), and serotype 29 (20/273, 7%), whereas 19% (53/273) of isolates remaining untypable. The isolates recovered from systemic sites of confirmed cases (SC) belonged to 14 serotypes with serotypes 2 (15%) and 9 (30%) as most prevalent, whereas the NSH isolates consisted of 18 serotypes with serotypes 2 and 9 together accounting for only 9% of isolates. However, 27% of isolates recovered from non-systemic sites of healthy pigs (NSH) were untypable compared with only 8.5% of untypable isolates in the SC group.

There were significant differences in the serotyping results by PCR and *in silico* serotyping (McNermar's test, *p* < 0.001, Supplementary Figure 3). PCR and *in silico* serotyping assigned the same serotype to an isolate at a rate of 72% (197/273). PCR could not distinguish 1/2 and 2, but 92% (22/24) of those isolates were classified into serotype 2 and 8% (2/24) as serotype 1/2 through *in silico* analysis. Of the remaining isolates, 6% were assigned to a different serotype by both methods and needed to be resolved by comparison of their *cps loci* to references with confirmed serotype. In addition, the number of isolates that were untypable by PCR was reduced by ~41% (87 to 53 isolates) through *in silico* serotyping. The PCR-untypable isolates were assigned by *in silico* into serotypes 2, 4, 8, 9, 15, 16, 21, 29, and 30. Most of the PCR-untypable isolates that were reclassified were found to be serotype 9 (9%, 8/87) and 21 (10%, 9/87).

### Relative Frequency of Virulence-Associated Factors Between Isolate Groups

**Figure 2:**
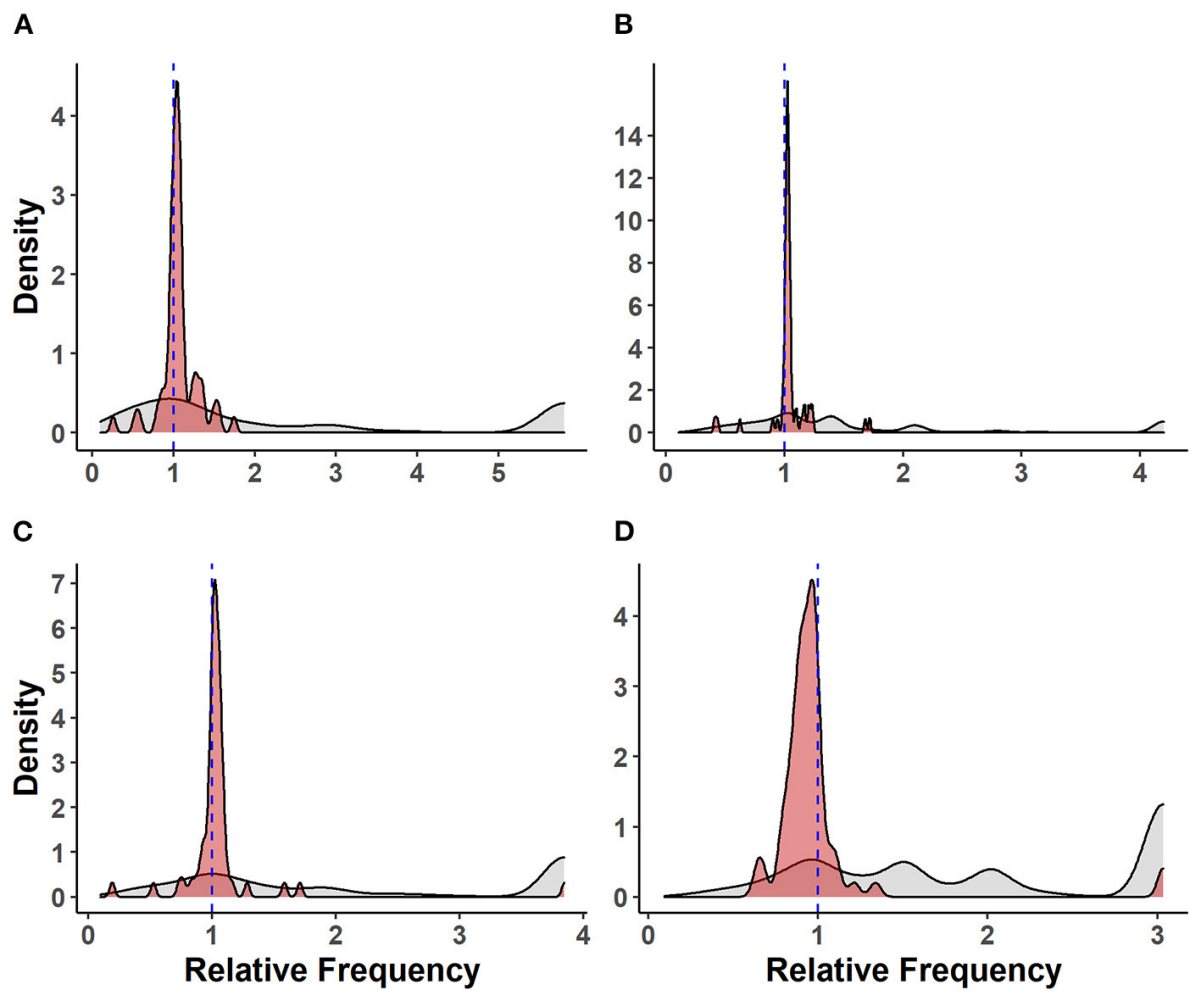
Distribution of virulence-associated factor genes and other genes carried by 273 *S. suis* isolates. Density plots of relative frequencies of known/putative VAF genes (red) against all other genes (gray) carried by each isolate. A relative frequency > 1 denotes a higher prevalence of gene within isolate group; **(A)** isolates recovered from systemic sites of pigs with confirmed infections (SC), **(B)** isolates recovered from non-systemic sites of pigs with confirmed infections (NSC), **(C)** isolates recovered from probable infections (NSP), **(D)** isolates recovered from non-systemic sites of healthy pigs (NSH).

The relative frequencies of VAF genes compared with other genes carried by *S. suis* isolates are shown in Figure 2. There was no significant difference between the mean relative frequencies of VAF genes in SC and NSC isolates [Kruskal–Wallis (KW), *n* = 112, *p* = 0.28]. However, there was an increased RF of VAF genes in both SC isolates (KW, *n* = 136, *p* < 0.001) and in NSC isolates (KW, *n* = 151, *p* < 0.001) compared with NSH isolates. Interestingly, the RF of VAF genes in NSP was also higher compared with NSH isolates (KW, *n* = 161, *p* < 0.001) but was not different from NSC isolates (KW, *n* = 161, *p* = 0.37).

### Relative Frequency of Virulence-Associated Factor Genes Relative to Non-virulence-associated Factor Genes Within Isolate Groups

There was a higher RF for VAF genes relative to other genes in SC (KW, *n* = 47, *p* < 0.001), NSC (KW, *n* = 65, *p* < 0.001), and NSP (KW, *n* = 71, *p* < 0.001) isolates. Conversely, there was a significant pattern of VAF genes having lower RF and being underrepresented in NSH isolates relative to non-VAF genes (KW, *n* = 90, *p* < 0.001).

### Frequency of Individual Virulence-Associated Factor Genes

The frequencies of individual VAF genes in 273 *S. suis* isolates in SC, NSC, NSP, and NSH are shown in Table 2 and Supplementary Table 2. Only three VAF genes including *dltA* [Fisher's test (FT), *p* < 0.001], *luxS* (FT, *p* = 0.011), and *troA* (FT, *p* = 0.019) had higher frequency in SC relative to NSH isolates. The *dltA, luxS*, and *troA* genes were present in 89, 96, and 96% of SC isolates, respectively, and these genes were further investigated using mixed-effect logistic regression method with farm and serotype as random effects (Supplementary Table 3). The gene *dltA* was found to have a higher probability of being present in SC (*p* < 0.001) and NSP (*p* = 0.010) isolates compared with NSH isolates. Compared with NSH isolates, the gene *luxS* was more likely to be present in NSP (*p* < 0.001) as well as in SC (*p* = 0.069) and NSC (*p* = 0.069) but borderline significant. Similar to *luxS*, the *troA* gene was also found to have a higher probability of being present in NSP isolates (*p* = 0.006) as well as a tendency to be increased in SC (*p* = 0.077) and NSC (*p* = 0.077) isolates. Serotype had a significant influence on the probability of each of the three genes being present (*p* < 0.001) within the isolate groups, but only the presence of the *dltA* gene was significantly influenced by the farm. Interestingly, all three genes were carried by all SC serotype 2 and 9 isolates, but not all NSH serotype 2 and 9 isolates carried the three genes. Of the three genes, *luxS* and *troA* were carried by all eight NSH serotype 2 isolates, and *dltA* was carried by two isolates. Among the six NSH serotype 9 isolates, 16% (1), 83% (5), and 83% (5) of isolates were carriers of *dltA, luxS*, and *troA*, respectively. Both *luxS* and *troA* generally had high rates of carriage by isolates regardless of the farm the isolate was recovered from. However, *dltA* was not as prevalent as *luxS* and *troA* and carried by 44% (84/190) of isolates on two farms—where mostly serotypes 9 and 2 isolates were recovered. Additionally, *dlta* had higher frequency in SC serotype 9 (FT, *p* < 0.001) and serotype 2 (FT, *p* < 0.025) isolates compared with their NSH counterparts.

**Table 2:** Distribution of individual virulence-associated factor (VAF) genes in 273 *S. suis* isolates in SC, NSC, NSP, and NSH groups.

**VAF/genes**	**Product**	**Number (%) of isolates**							
		**Total** **(*n* = 273)**	**SC** **(*n* = 47)**	**NSC** **(*n* = 65)**	**NSH** **(*n* = 90)**	**NSP** **(*n* = 71)**	**Ser 9** **(*n* = 38)**	**Ser 2** **(*n* = 23)**	**Unt** **(*n* = 53)**
*AgI/II*	Antigen I/II	70 (26)	16 (34)	23 (35)	24 (27)	7 (10)	23 (61)	0 (0)	8 (15)
*apuA*	Amylopullulanase	266 (97)	46 (97)	64 (98)	87 (97)	69 (97)	38 (100)	23 (100)	48 (90)
dltA^*^	D-alanine–D-alanyl carrier protein ligase	190 (70)	42 (89)	46 (71)	53 (59)	49 (69)	21 (55)	15 (65)	25 (47)
*epf*	Extracellular protein factor	27 (10)	5 (11)	4 (6)	6 (7)	12 (16)	0 (0)	0 (0)	2 (4)
*guaA*	GMP synthase	262 (96)	44 (94)	64 (98)	85 (94)	69 (97)	38 (100)	23 (100)	46 (87)
luxS^*^	S-ribosylhomocysteine lyase	243 (89)	45 (96)	59 (91)	71 (79)	68 (96)	37 (97)	23 (100)	34 (64)
*mrp*	Muramidase-released protein	65 (24)	15 (32)	19 (29)	17 (19)	14 (20)	0 (0)	15 (65)	2 (4)
*pgdA*	Peptidoglycan N-deacetylase	245 (90)	44 (94)	60 (92)	74 (82)	67 (94)	37 (97)	23 (100)	34 (64)
*sly*	Suilysin	77 (28)	13 (28)	20 (31)	22 (24)	22 (31)	1 (2)	0 (0)	4 (8)
troA^*^	Metal binding lipoprotein TroA	245 (90)	45 (96)	60 (92)	73 (81)	67 (94)	37 (97)	23 (100)	34 (64)

*SC = isolates from systemic sites in pigs with confirmed infections; NSC = isolates from non-systemic sites in pigs with confirmed infections; NSP = isolates from non-systemic sites in symptomatic pigs, which had no S. suis recovered from their systemic sites; NSH = isolates from non-systemic sites in healthy pigs; Total = SC + NSC + NSH + NSP*.

* *Higher frequency in SC compared with NSH (p < 0.05)*.

### Muramidase-Released Protein, Suilysin, Extracellular Factor Genotypes

The *mrp, sly, epf*, and *epf* ^*^ gene were carried by 24% (66), 28% (69), 10% (27), and 7% (18) of 273 isolates, respectively (Table 3). None of the serotype 2 isolates carried *epf* or *sly* genes. Furthermore, all the serotype 9 isolates except for one *mrp*^+^ isolate belonged to the *mrp*^−^*sly*^−^*epf*^−^ genotype. Overall, 62% (168/273) of the isolates, 58% (106/183) of SC, NSC, and NSP isolates collectively, and 69% (62/90) of NSH isolates belonged to the *mrp*^−^*sly*^−^*epf* ^−^ genotype. The *mrp*^+^*sly*^+^*epf*^+^ and *mrp*^+^*sly*^+^epf^*^+ genotypes were only identified in 8% (21/273) and 2% (5/273) of the isolates, respectively. Twenty-four isolates with *mrp*^+^*sly*^+^*epf*^+^/epf^*^+ genotypes belonged to serotypes 3, 4, 7, 11, 15, 28, 30, and 31; two untypable NSC isolates (recovered from rectal samples) had the *mrp*^+^*sly*^+^*epf*^+^ genotype. Nine of these 24 isolates with *mrp*^+^*sly*^+^*epf*^+^ / epf^*^+ genotypes (serotypes 3, 7, 11, 28, and 30) were recovered from non-systemic sites of healthy pigs (NSH).

**Table 3:** *S. suis* genotypes based on classical virulence markers (40, 42–44).

**Genotype**	**Number (%) of Isolates**																
	**All (*****N*** **=** **273)**					**Ser 9 (*****n*** **=** **38)**				**Ser 2 (*****n*** **=** **23)**				**Unt (*****n*** **=** **53)**			
	**T**	**SC**	**NSC**	**NSP**	**NSH**	**SC**	**NSC**	**NSP**	**NSH**	**SC**	**NSC**	**NSP**	**NSH**	**SC**	**NSC**	**NSP**	**NSH**
*mrp^**−**^sly^**−**^epf^**−**^*	168 (62)	23 (49)	38 (58)	45 (63)	62 (69)	14 (100)	9 (100)	9 (100)	5 (83)	–	1 (50)	2 (33)	5 (50)	4 (100)	7 (78)	14 (88)	24 (100)
*mrp^**+**^sly^**−**^epf^**−**^*	28 (10)	11 (23)	7 (11)	4 (6)	6 (7)	–	–	–	–	7 (100)	1 (50)	4 (67)	3 (50)	–	–	–	–
*mrp^**−**^sly^**+**^epf^**−**^*	21 (8)	2 (4)	5 (8)	8 (11)	6 (7)	–	–	–	1 (17)	–	–	–	–	–	–	2 (13)	–
*mrp^**+**^sly^**+**^epf^**+**^*	21 (8)	2 (4)	4 (6)	9 (13)	6 (7)	–	–	–	–	–	–	–	–	–	2 (22)	–	–
mrp^**−**^sly^**+**^epf^*^^+^	13 (5)	4 (8)	3 (5)	1 (1)	5 (6)	–	–	–	–	–	–	–	–	–	–	–	–
*mrp^**+**^sly^**+**^epf^**−**^*	11 (4)	2 (4)	6 (9)	1 (1)	2 (2)	–	–	–	–	–	–	–	–	–	–	–	–
*mrp^**−**^sly^**+**^epf^**+**^*	6 (2)	2 (4)	–	3 (4)	–	–	–	–	–	–	–	–	–	–	–	–	–
mrp^**+**^sly^**+**^epf^*^^+^	5 (2)	–	2 (3)	–	3 (3)	–	–	–	–	–	–	–	–	–	–	–	–
Total	273	47	65	71	90	14	9	9	6	7	2	6	8	4	9	16	24

–epf^*^ = large variant extracellular factor protein; Ser, Serotype; Unt, Untypables; SC = isolates from systemic sites in pigs with confirmed infections; NSC = isolates from non-systemic sites in pigs with confirmed infections; NSH = isolates from non-systemic sites in healthy pigs; NSP = isolates from non-systemic sites in symptomatic pigs, which had no S. suis recovered from their systemic sites; Percentage in each column (per subset) is out of the total number indicated in the last row.

### Virulence-Associated Factor Profiles of *S. suis* Serotypes

**Figure 3:**
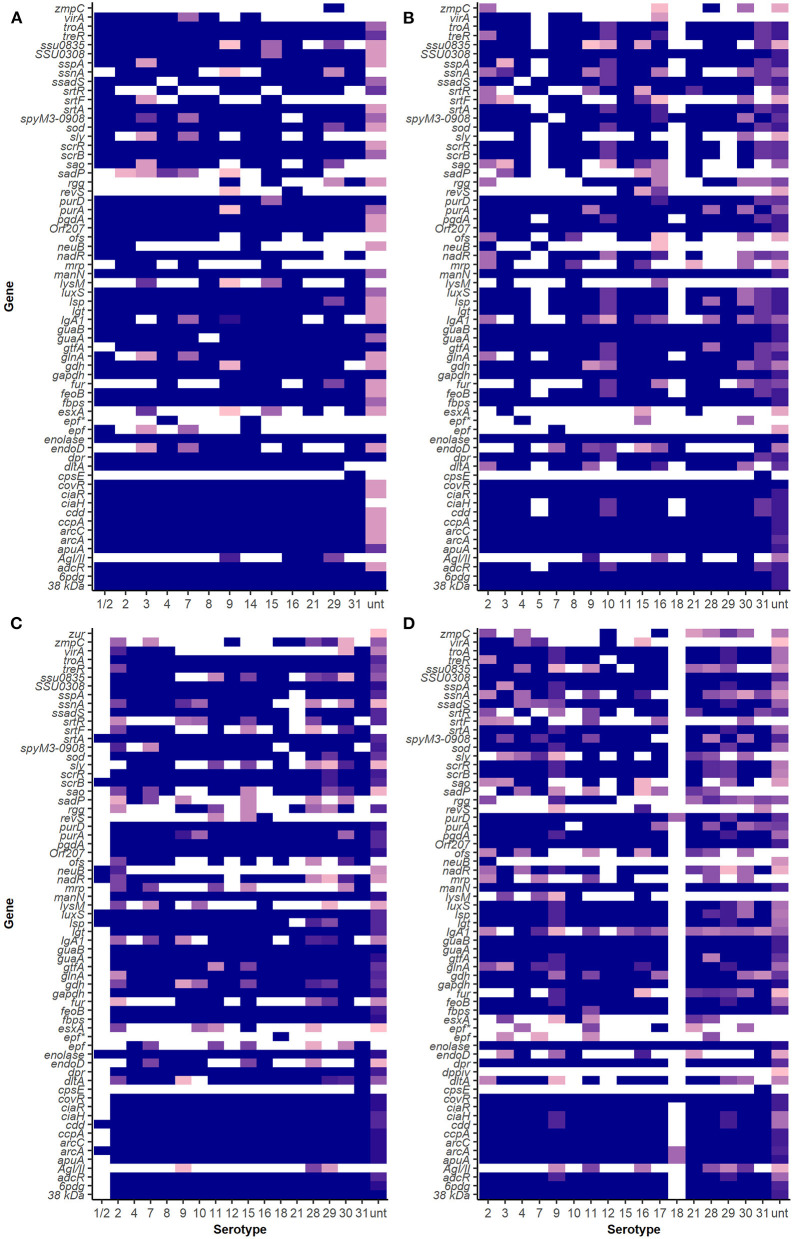
Virulence-associated factor gene distribution among isolates in SC, NSC, NSP, and NSH groups. Darker shades show a higher proportion of isolates of a serotype carrying a particular gene. White indicates an absence of gene in that serotype. **(A)** Isolates recovered from systemic sites of pigs with confirmed infections (SC), **(B)** isolates recovered from non-systemic sites of pigs with confirmed infections (NSC), **(C)** isolates recovered from probable cases (NSP), and **(D)** isolates recovered from non-systemic sites of healthy pigs (NSH).

**Figure 4:**
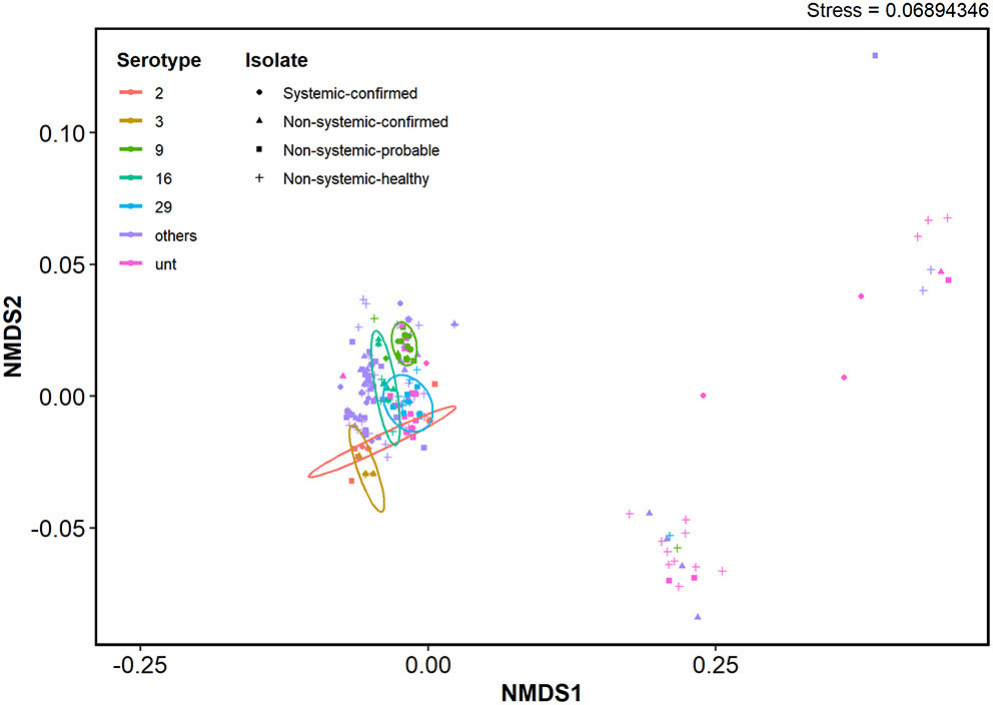
NMDS clustering based on virulence-associated factor gene presence and absence profiles of *S. suis* isolates. Ordination was based on a Jaccard distance matrix generated using VAF gene presence and absence data. Ellipses represent clusters within a 95% confidence level for each isolate group. Serotypes with prevalence below 5% were grouped together as “other”.

The VAF gene profiles in serotypes 2 and 9 in SC, NSC, NSP, and NSH isolate group are demonstrated in Figure 3 and Supplementary Figures 4, 5. Clustering analysis using NMDS of VAF genes presence and absence data showed that most typable isolates clustered together (Figure 4). The serotype 2, 3, 9, 16, and 29 isolates formed their own clusters within the cluster of typeable isolates, and there were a few divergent strains that were mostly untypable isolates.

Of 53 different VAF genes identified in serotype 9 isolates in the SC group, only 45 of those genes were carried by all isolates. All except one serotype 2 isolate in the SC group carried the same 51 VAF genes. There were some VAF genes that were carried only by serotype 2 and not serotype 9 isolates in the SC group, which included the genes *virA, srtF, sadP, ofs, neuB, mrp*, and *IgA1*. Similarly, *srtR, rgg, lysM, fur, esxA, endoD*, and *AgI/II* were identified in serotype 9 isolates in the SC group but were not found in serotype 2 isolates in the SC group.

The *srtR* gene was found in 13% of serotype 9 and was also found in some isolates of 15 other different serotypes in the SC isolate group. The gene *AgI/II* was only found in serotype 9 (75%, 12/16) and two serotype 29 isolates among the SC isolate but was also found in other serotypes (9, 16, 21, 28, 29, and 30) in the NSC and NSH isolate groups (Table 2). Interestingly, *lysM* and *esxA* were rarely found in SC serotype 9 (5%, 1/21) and were found in 6 (3, 7, 8, 9, 10, and 16) and 8 (3, 9, 10, 11, 15, 21, 28, and unt) serotypes, respectively, in the NSC and NSH isolate groups. The gene *ssadS* was identified in isolates belonging to all 22 serotypes (87%, 238/273). Among the SC isolates, *ssadS* was detected in all serotype 9 (14) and serotype 2 (7) isolates and was also detected in 67% (4/6) and 100% (8) of NSH serotype 9 and 2 isolates, respectively.

### Multilocus Sequence Typing

The serotype 2 isolates in this study were distributed among seven different STs. All serotype 2 isolates in the SC group, one isolate in the NSC group, and three isolates in the NSH group were determined to be ST25, which explains their similar VAF profiles (Supplementary Figure 6). However, the remaining serotype 2 isolates in the NSC, NSP, and NSH groups belonged to six different STs—three isolates in the NSP group were ST28, and the rest of the isolates from the NSC and NSH groups were assigned to five distinct novel STs. There was only in one pig where ST25 was recovered from both systemic and non-systemic sites. In addition, the three ST25 in the NSH isolate group were isolated from pigs on the same farms that the SC ST25 isolates were recovered from. The serotype 9 isolates showed higher diversity with isolates belonging to 13 novel STs and ST621. Among the serotype 9 isolates, 12 of 14 isolates in the SC group and 5 of 11 isolates in the NSC group belonged to one novel ST; the remaining two isolates in the SC group were assigned to two other novel STs, one NSH isolate was identified as ST621, and the rest, along with other isolates in the NSC, NSH and NSP groups, were spread among 12 novel STs. Five isolates sharing the same ST among the SC and NSC groups were recovered from the same respective pigs.

### Antimicrobial Resistance

**Figure 5:**
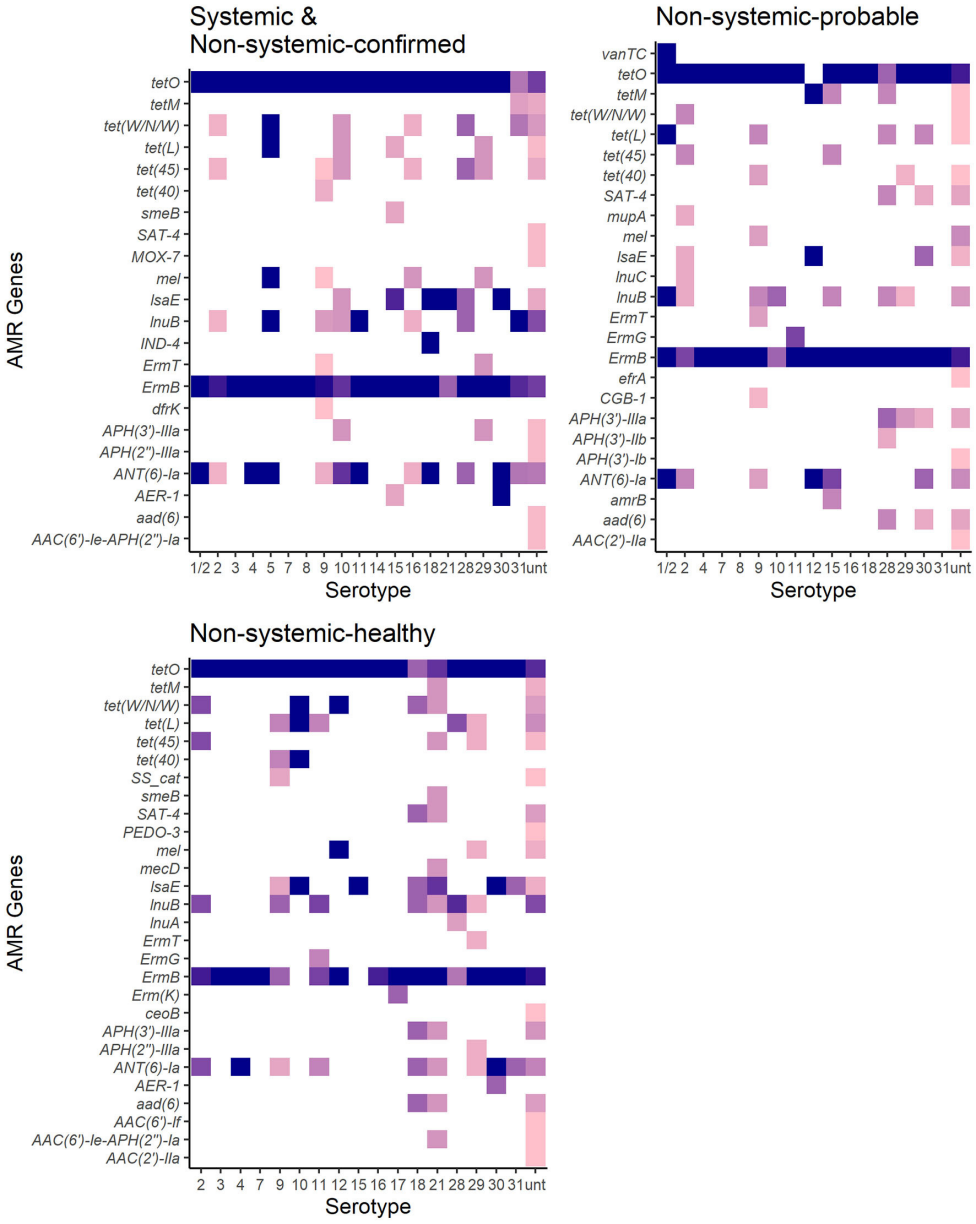
Antimicrobial resistance genes distribution in 267 *S. suis* isolates from SC and NSC and NSP and NSH groups. Darker shades show a higher proportion of isolates of serotypes carrying a particular gene. White indicates an absence of gene in that serotype.

There were 38 different genes encoding resistance to 15 antibiotic drug classes detected in 273 *S. suis* isolates (Figure 5, Table 4). Overall, 98% (267/273) of isolates carried at least one AMR gene, and 79% (217/273) of isolates carried genes associated with resistance to at least four drug classes (Supplementary Tables 5, 6). The four major drug classes to which resistance genes were found included tetracycline in 98% (267/273), lincosamides in 96% (263/273), macrolide in 90% (247/273), and streptogramin in 90% (247/273) of isolates. The *mel* or *IsaE* gene encoding resistance to pleuromutilin antibiotics was identified in 20% (47/273) of isolates. The genes encoding resistance to penam and cephalosporin were detected only in 2.6% (7/273) and (1.7% (4/273), respectively.

**Table 4:** AMR genes detected in 273 *S. suis* isolates in SC, NSC, NSP, and NSH groups.

**Drug Class**	**Number (%) of isolates**					**Genes/Proteins**
	**Total** **(*n* = 273)**	**SC** **(*n* = 47)**	**NSC** **(*n* = 65)**	**NSP** **(*n* = 71)**	**NSH** **(*n* = 90)**	
Aminoglycosides	111 (41)	10 (21)	23 (35)	35 (49)	43 (48)	*ANT(6)-Ia, AAC(2′)-IIa, aad(6), APH(3′)-IIIa, AAC(6′)-Ie-APH(2″)-Ia, amrB, AAC(6′)-If, APH(3′)-IIb, APH(3′)-Ib, APH(2″)-IIIa*
Carbapenems	3 (<1)	1 (1)	1 (1)	0 (0)	1 (1)	*PEDO-3,IND-4*
Diaminopyrimidines	1 (<1)	0 (0)	1 (1)	0 (0)	0 (0)	*dfrK*
Glycopeptides	1 (<1)	0 (0)	0 (0)	1 (1)	0 (0)	*vanTC*
Lincosamides	72 (26)	7 (15)	17 (26)	15 (21)	33 (37)	*lnuB, lnuC*
Mupirocin	1 (<1)	0 (0)	0 (0)	1 (1)	0 (0)	*mupA*
Nucleosides	14 (<1)	0 (0)	1 (1)	6 (8)	7 (8)	*SAT-4*
Penams	4 (<1)	0 (0)	2 (2)	0 (0)	2 (2)	*mecD, AER-1*
Phenicols	2 (<1)	0 (0)	0 (0)	0 (0)	2 (2)	*SS_cat*
Tetracycline	267 (98)	47 (100)	63 (97)	70 (99)	87 (97)	*tetO, tet(45), tet(W/N/W), tetM, tet(L), tet(40)*
**MULTIDRUG RESISTANCES**						
Aminoglycoside, cephalosporin, cephamycin, penam	2 (<1)	1 (1)	0 (0)	0 (0)	1 (1)	*smeB*
Carbapenem, cephalosporin, penam	1 (<1)	0 (0)	0 (0)	1 (1)	0 (0)	*CGB-1*
Cephalosporin, cephamycin, penam	1 (<1)	1 (1)	0 (0)	0 (0)	0 (0)	*MOX-7*
Fluoroquinolone, aminoglycoside	1 (<1)	0 (0)	0 (0)	0 (0)	1 (1)	*ceoB*
Macrolide, fluoroquinolone, rifamycin	1 (<1)	0 (0)	0 (0)	1 (1)	0 (0)	*efrA*
Macrolide; lincosamide; streptogramin (MLS)	245 (90)	47 (100)	54 (83)	69 (97)	75 (83)	*ermB, ermG, ermT, ermK*
MLS, tetracycline, oxazolidinone, phenicol, pleuromutilin	57 (<1)	4 (9)	18 (28)	14 (20)	19 (21)	*mel, IsaE*

*SC = isolates from systemic sites in pigs with confirmed infections; NSC = isolates from non-systemic sites in pigs with confirmed infections; NSP = isolates from non-systemic sites in symptomatic pigs, which had no S. suis recovered from their systemic sites; NSH = isolates from non-systemic sites in healthy pigs*.

There was no significant difference between the RF of AMR genes carried by isolates in the SC and NSC groups (KW, *n* = 112, *p* = 1.00). However, there was a higher RF of AMR genes in NSH isolates compared with SC and NSC isolates, collectively (KW, *n* = 202, *p* = 0.008). Furthermore, there were 28 AMR genes identified in NSH isolates compared with 22 AMR genes in SC and NSC isolates, collectively (Figure 5). There were seven genes found in NSH isolates that were absent in SC and NSC isolates, including *cat* (2 isolates, chloramphenicol resistance), *PEDO-3* (1 isolate, carbapenem resistance), *ermG* (1 isolate, MLS), *ermK* (1 isolate, MLS), *ceoB* (1 isolate, fluoroquinolone and aminoglycoside resistance), *AAC(6*′*)-If* (1 isolate, aminoglycoside resistance), and *AAC(2*′*)-IIa* (1 isolate, aminoglycoside resistance). There were eight AMR genes present in >20% of isolates (Table 5). Most of the prevalent AMR genes had similar distributions across all four isolate groups; however, the *ermB* gene encoding MLS resistance was detected in higher frequency in SC isolates (98%) than in NSH isolates (81%) (FT, *p* = 0.009). Interestingly, all the serotype 2 isolates carried genes encoding resistance to tetracycline (*tetO*). Serotype 2 isolates in the NSH group also carry other genes such as *tet(W/N/W)* and *tet(45)* encoding tetracycline resistance.

**Table 5:** Distribution and frequency of most prevalent antimicrobial resistance genes in 273 *S. suis* isolates in SC, NSC, NSP, and NSH groups.

**AMR Genes**	**Drug Class**	**Number (%) of isolates**							
		**Total** **(*n* = 273)**	**SC** **(*n* = 47)**	**NSC** **(*n* = 65)**	**NSH** **(*n* = 90)**	**NSP** **(*n* = 71)**	**Ser 9** **(*n* = 38)**	**Ser 2** **(*n* = 23)**	**Unt** **(*n* = 53)**
ermB^*^	Macrolide; lincosamide; streptogramin	237 (87)	46 (98)	53 (82)	73 (81)	65 (92)	34 (89)	19 (83)	45 (85)
*APH(3′)-IIIa*	Aminoglycosides	20 (7)	1 (2)	2 (3)	8 (9)	9 (13)	0 (0)	0 (0)	10 (19)
*tet(L)*	Tetracycline	29 (11)	2 (4)	3 (5)	15 (17)	9 (13)	5 (13)	0 (0)	9 (17)
lnuB^**^	Lincosamides	70 (27)	7 (15)	17 (26)	32 (36)	14 (20)	11 (29)	7 (30)	27 (51)
*tetO*	Tetracycline	247 (91)	46 (98)	57 (88)	80 (89)	64 (90)	38 (1)	23 (100)	42 (79)
*lsaE*	Pleuromutilin; lincosamides; streptogramin	38 (14)	3 (6)	14 (22)	14 (16)	7 (10)	1 (3)	1 (4)	7 (13)
*tet(W/N/W)*	Tetracycline	27 (10)	2 (4)	8 (12)	14 (16)	3 (4)	0 (0)	8 (35)	9 (17)
*ANT(6)-Ia*	Aminoglycosides	66 (23)	7 (15)	20 (31)	23 (26)	16 (8)	6 (16)	8 (35)	18 (34)

*SC = isolates from systemic sites in pigs with confirmed infections; NSC = isolates from non-systemic sites in pigs with confirmed infections; NSH = isolates from non-systemic sites in healthy pigs; Total = SC + NSC + NSH + NSP*.

* *Higher frequency in SC compared with NSH (p < 0.05)*.

** *Higher frequency in NSH compared with SC (p < 0.05)*.

**Figure 6:**
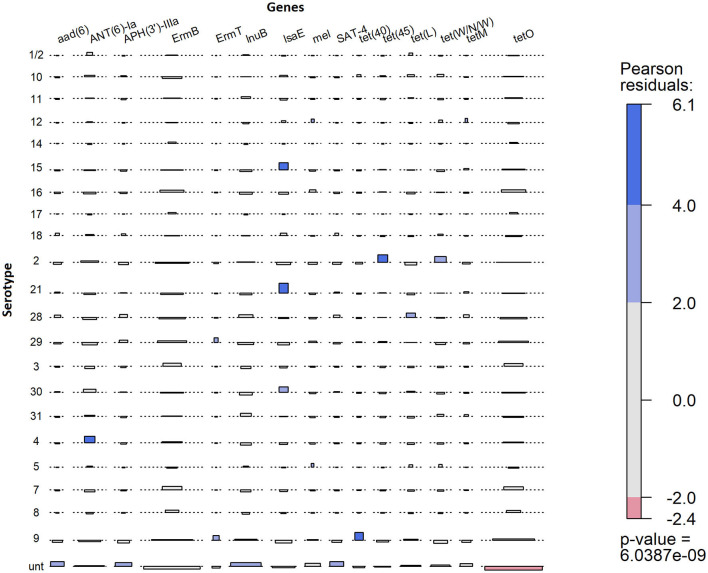
Association plot for presence of AMR genes in 273 *S. suis* isolates belonging to different serotypes. Generated using Pearson's chi-squared test of independence of multi-way contingency table. Shade represents a degree of departure from expected values; blue for higher and red for lower.

The observed proportion of isolates that carries AMR genes *tet(45), tet(40), lsaE* and *ANT(6)Ia, tet(W/N/W), ermT*, and *tetO* was different than expected within specific serotypes (Figure 6). The only AMR gene found to be underrepresented was *tetO* in untypable isolates. The genes *tet(45)* and *tet(W/N/W)* were each found in 2 (9%) serotype 2 isolates. The genes *tet(40)* and *ermT* were identified in 18% (7/38) and 8% (3/38) of serotype 9 isolates. However, most serotype 9 isolates did not carry these two genes, and therefore, the genes are not associated with serotype 9. The presence of *lsaE* was positively associated with serotype 21 [Chi-squared (*X*^2^), *p* = 0.011]; however, 83% (5/6) of the serotype 21 isolates carrying *lsaE* were from the same farm. Similarly, the presence of *ANT(6)-Ia* was positively associated with serotype 4 isolates (*X*^2^, *p* = 0.020), recovered from the same farm.

## Discussion

This study used WGS data to type *S. suis* isolates and examine the association of disease status and the presence of VAF and AMR genes in a collection of isolates recovered from sick and healthy pigs. There are numerous VAFs reported with a possible association with *S. suis* disease development (40, 45). However, there have not been many studies that investigated the distribution of these VAFs within a large collection of *S. suis* isolates from sick and healthy pigs.

In this study, WGS data were used for serotyping *S. suis* isolates using *in silico* method. The classification of *S. suis* isolates is key to the development of control and prevention measures of *S. suis* disease in pigs. *S. suis* serotyping can be done through multiple methods, including slide co-agglutination test (29), PCR serotyping (30–33), and *in silico* (34). The present study compared PCR with *in silico* serotyping and found a lack of agreement between the two methods. The observed disagreement was mostly attributed to the differentiation of serotypes 1/2 and 2 by *in silico* method, as well as serotyping of more than 40% of PCR-untypable isolates. The inability of PCR to distinguish between serotypes 1 and 14, and 2 and 1/2 is a result of the high similarity within the *cps* gene clusters of the two pairs of serotypes (31, 70). Furthermore, PCR fails to serotype some strains potentially because they carry a novel *cps* gene cluster or a mutant *cps* gene cluster (30, 71). Similar to PCR, the slide co-agglutination test also does not distinguish between pairs of serotypes due to shared antigenic determinants (5) and cannot classify isolates with novel CPS (72). However, *in silico* serotyping is able to distinguish between serotypes 1 and 14 and 2 and 1/2 and classified untypable strains by directly comparing serotype-specific SNPs within the *cps* sequences (34). Recently, a new PCR-based technique called mismatch amplification mutation assay (33) has been developed targeting these SNPs to discriminate between serotypes 1 and 14 and 2 and 1/2. Therefore, except for untypable isolates, *S. suis* can now be accurately classified through combinations of multiple PCR assays. Both PCR and *in silico* typing methods are therefore a great resource when working with high number of isolates. Additionally, in comprehensive studies, *in silico* serotyping may be able to provide more information by typing the strains that could not be serotyped by PCR or slide co-agglutination method. It is important to keep in mind, however, that the *in silico* pipeline by Athey et al. (34) may not identify all novel isolates recovered since 2015.

There are numerous VAFs characterized and reported to play important roles in *S. suis* virulence (40, 45). The present study found a tendency for *S. suis* isolates recovered from sick pigs to carry a higher number of VAF genes, which is in accordance with previous analysis of frequencies of the same set of VAFs in *S. suis* isolates conducted by Weinert et al. (24). Additionally, there was no significant difference between the carriage of VAFs by isolates from systemic and non-systemic sites of pigs with confirmed infections. This indicates the potential of isolates recovered from non-systemic sites to cause systemic infection. In line with this observation, it was previously described that some carrier piglets develop clinical signs due to dissemination of *S. suis* from non-systemic sites such as tonsils and other mucosal surfaces to systemic sites (1, 73). The higher frequency of VAFs in NSC isolates relative to NSH isolates could indicate that there are environmental or host-associated factors that benefit isolates carrying these genes and, therefore, could lead to a higher likelihood of disease development. However, as the NSH isolates in the current study were obtained from the same farms as the sick pigs, farm-level environmental or management factors may be less of a factor than animal-level variations for pathogen success. For instance, we detected ST25 strains in isolates from healthy pigs on the same farm where ST25 strains were detected in isolates from systemic sites of sick pigs indicating that host- and environment-associated factors are likely involved in disease development. Further epidemiological studies are required before any concrete conclusions can be made.

This present study also determined that most of the individual VAFs did not have a significant correlation with disease status despite the higher RF of VAFs in sick animals. It is possible that VAFs generally increase the fitness of *S. suis* strains and are favored for colonization but are not necessarily causing the illness, or it could indicate that combinations of different VAFs can contribute to disease development. The VAF genes with significantly higher frequencies in SC isolates in this study were *dltA, luxS*, and *troA*. Similarly, a previous study on virulent serotype 9 and 2 strains from Spain and Canada also reported that *dltA* and *luxS* were present in all 30 isolates analyzed (7). All the genes detected in higher frequencies play key roles in environmental adaptation and survival in *S. suis*. *LuxS* plays critical roles in quorum sensing and regulation of expression of other VAFs (74). *TroA* is involved in the uptake of manganese used by *S. suis* for counteracting oxidative stress (75). The gene *dltA* is involved in the survival of *S. suis* in blood, as it plays a role in escaping neutrophil-mediated immune responses (76). The gene *dltA* has also been found to play key roles in the virulence of other Gram-positive bacteria, including *Streptococcus agalactiae* (77), *Streptococcus aureus* (78), *Streptococcus pyogenes* (79), and *Listeria monocytogenes* (80). Further studies aimed at characterizing and understanding the specific advantages that these three VAFs provide in *S. suis* pathogenesis would be useful.

The three virulence markers including *mrp, epf*, and *sly* have previously been used as indicators of high virulence in serotype 2 strains (45). The detection of *mrp* in all serotype 2 isolates from systemic sites in the current study supports the previous finding that *mrp* may play a role in adherence of serotype 2 strains to host cells and survival in the blood (41). In the present study, more than half of the isolates recovered from systemic isolates and all serotype 9 strains from healthy and sick pigs were *mrp* negative. Fittipaldi et al. (38) have also shown the absence of *mrp* in serotype 2 isolates recovered from pigs with clinical disease from Canada and the United States. The absence of *mrp* was also observed for most serotype 9 strains in sick pigs in previous studies (69, 81). The absence of *mrp* in *S. suis* isolates recovered from sick pigs supports the explanation that VAFs may be favored for their roles in colonization but not necessarily in causing disease (82).

Similar to the current study, previous analyses of serotype 2 and 9 strains also noted rare detection or absence of *epf* (7, 69, 81). Notably, none of the serotype 2 and 9 isolates in this study carried either *sly* or *epf*, although these genes are involved in important virulence mechanisms in *S. suis*. Although not essential, SLY is very important in the invasion and survival of *S. suis* in its host (83). The absence of this and other classical markers in most isolates suggests that the virulence potential of the *S. suis* isolates in this study is likely lower than isolates from other countries such as Spain, where a higher prevalence of *sly* and *mrp* has been reported (7). Our results mirror that of a recent study on pathogenic indicators in *S. suis* in the United States—where they have also found that these three classical markers are not strong predictors of pathogenic *S. suis* isolates (84). The absence of important VAFs in some isolates from systemic sites in the present study may highlight that many VAFs play similar roles (40). The presence of all three markers in isolates recovered from healthy pigs in this study may also suggest that important VAFs are not exclusive to pathogenesis but likely play other essentials roles in the protection and propagation of *S. suis*. Furthermore, just presence of VAFs genes may not be sufficient to develop the disease, and it needs to be accompanied by other factors such as farm management, diet, pig flow, pig density, and presence of other diseases. Other factors such as host genetics and interactions with other members of the microbiota may also have important influences in the virulence mechanisms of each serotype, and each may have serotype-specific genetic determinants (85). Alternatively, it might also be possible that these genes in healthy isolates are not expressed.

It has previously been shown that serotype 9 isolates were generally less virulent compared with serotype 2 isolates (86, 87). This suggests that there might also be differences in the genetic bases of their virulence. In this study, serotype 9 and 2 isolates from systemic sites carried additional VAFs that were distinct from each serotype. For example, *srtR* and *AgI/II*, which were predominantly present in SC serotype 9 isolates in this study, have previously been reported as important VAFs in serotype 9 pathogenesis. *SrtR* was reported to be involved in stress tolerance (48), and *AgI/II* (50) was reported to be involved in the survival and development of systemic disease. Furthermore, in our NMDS analysis, isolates from the most prevalent serotypes formed clusters within a bigger cluster of typable isolates indicating similarities in VAF genes carried among the well-characterized serotypes despite differences in gene carriage for individual serotypes. This supports the possibility that virulence mechanisms of each serotype may have slight differences, but there are not enough representative isolates within each serotype in the current study to determine distinct sets of VAFs per serotype. The recent increase in the prevalence of disease caused by serotype 9 isolates globally requires more attention to understanding the pathogenesis mechanisms that may be unique to each serotype.

There was a high rate of carriage of AMR genes in *S. suis* isolates in this study, and similar to previous *S. suis* genotypic AMR studies globally (20, 22, 23), most of the isolates carried genes associated with resistance to tetracycline, lincosamides, and macrolides. Consistent with those studies, *tet(O)* was the most prevalent tetracycline resistance gene detected in the *S. suis* isolates. In the present study, *tet(45)* and *tet(W/N/W)* were identified in several serotypes, including several serotype 2 isolates, which have not been previously described. However, *tetM*, which was frequently associated with *S. suis* serotype 2 strains from patients in China and Vietnam (88, 89) was absent in all serotype 2 strains in this study. Previous studies have also demonstrated that clindamycin and chloramphenicol resistance was associated with serotype 2 (90). In the present study, none of the serotype 2 isolates carried genes associated with chloramphenicol resistance, whereas the genes associated with lincosamides resistance were common in all serotypes and not just serotype 2. In this study, AMR genes were found in higher diversity and frequency in healthy pigs compared with isolates from sick pigs. The difference in frequency within the *S. suis* collection of this study may be attributed to the higher diversity of isolates detected within healthy pigs. The SC isolates consisted mostly of genotypically related isolates, particularly those of serotypes 2 and 9, with each group of related isolates carrying a similar set of AMR genes. The presence of some AMR genes was statistically associated with specific serotypes, but it was more likely seen in a group of isolates recovered from pigs on the same farm. Previous findings suggest that resistance patterns in *S. suis* can vary with pig health, geographic location, serotypes, and different farm practices such as the use of different antibiotics (8, 10), which likely contributed to the presence of the same AMR genes on a specific farm. Notably, in the current study, the isolates were not evaluated for phenotypic resistance, and therefore, further studies are needed to compare the presence of AMR genes in *S. suis* isolates with confirmed phenotypic resistance.

This study has shown that *in silico* serotyping can improve the classification of *S. suis* and its contributions in comprehensive genomic *S. suis* studies. It also highlighted that the presence of VAFs is not the sole indicator of disease development. A large number of *S. suis* serotypes and the prevalence of many of them in clinical infections globally increase the importance of understanding the differences and, more importantly, the shared factors involved in the pathogenesis of each serotype—in developing a universal solution. The results in this study showed differences in virulence factors between serotypes, and there is a need to expand research on the virulence mechanism of clinically prevalent *S. suis* serotypes other than serotype 2 and develop a more holistic understanding of *S. suis* pathogenesis. With such considerations, a future study incorporating a much larger global data set to investigate shared genetic determinants and other factors should be very interesting and insightful in efforts such as vaccine development. The high frequency of AMR genes in this study suggests that consistent surveillance of AMR in *S. suis* is crucial. Future studies on the potential mobility of these AMR genes among *S. suis* and other bacterial populations are imperative in the infection control efforts. Further research into the distribution patterns of VAF and AMR genes may give insight into the development of methods and guidelines for *S. suis* control.

## Data Availability Statement

The datasets presented in this study can be found in online repositories. The names of the repository/repositories and accession number(s) can be found at: https://www.ncbi.nlm.nih.gov/, PRJNA754245.

## Ethics Statement

The animal study was reviewed and approved by University of Guelph Animal Care Committee.

## Author Contributions

AF, NR, and ZP conceived and developed the study designed. MA performed data analysis and wrote the paper. NR, AF, ZP, and NF offered their expertise and helped guide the development of the study. All authors have read, reviewed, edited, and approved the manuscript.

## Funding

This work was supported by Ontario Pork (OP19-005), Ontario Ministry of Agriculture, Food and Rural Affairs (UofG2018-3122), Swine Innovation Porc (1794b), and Canada First Research Excellence Fund, Food from Thought (499105).

## Conflict of Interest

The authors declare that the research was conducted in the absence of any commercial or financial relationships that could be construed as a potential conflict of interest.

## Publisher's Note

All claims expressed in this article are solely those of the authors and do not necessarily represent those of their affiliated organizations, or those of the publisher, the editors and the reviewers. Any product that may be evaluated in this article, or claim that may be made by its manufacturer, is not guaranteed or endorsed by the publisher.
