# Supplementary material for: Serotypes, Virulence-Associated Factors, and Antimicrobial Resistance of Streptococcus suis Isolates Recovered From Sick and Healthy Pigs Determined by Whole-Genome Sequencing
Source: Front Vet Sci. 2021 Nov 2;8:742345. doi: 10.3389/fvets.2021.742345 (PMC8593187; doi:10.3389/fvets.2021.742345)
Supplement: Supplementary file 1 [file Data_Sheet_1.PDF]

# Supplemental Data

## 1.1 Supplementary Figures

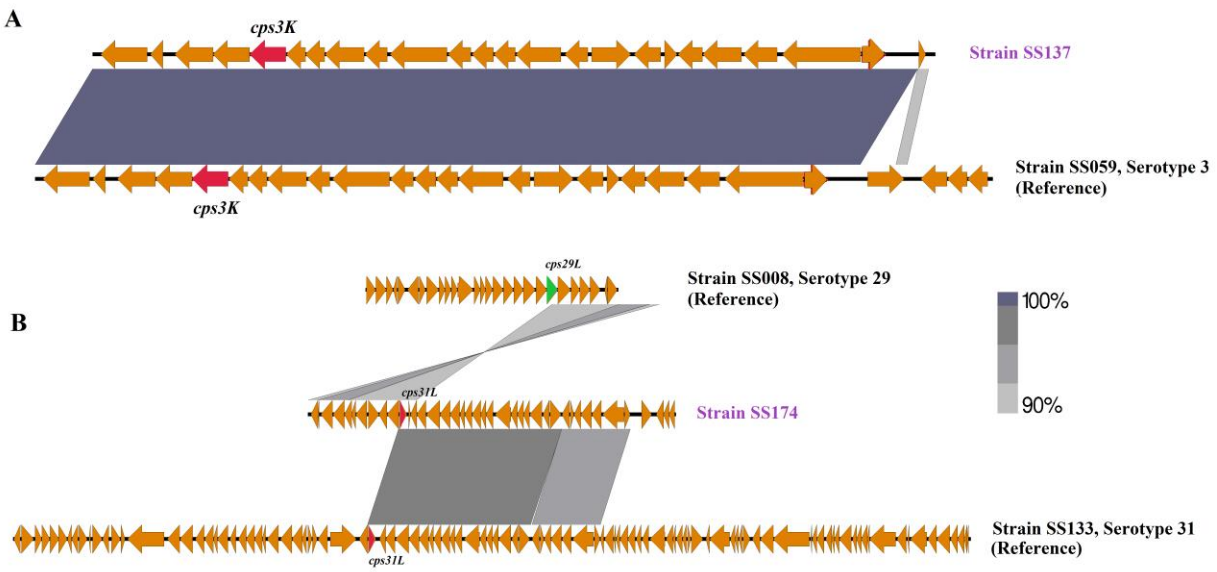


**S.D. Fig S1: Example confirmation of serotypes when strains are assigned mismatched serotypes by PCR and *in silico* serotyping**. The cps loci of strains assigned mismatched serotypes were compared to references with confirmed serotypes using the program easyFig. (**A**) Strain SS137 was classified as serotype 18 by PCR serotyping, but *cps* loci comparison shows it is serotype 3, as assigned by *in silico* serotyping. (**B**) Strain SS174 was classified as serotype 29 by PCR serotyping, but *cps* loci comparison shows it is serotype 31, as assigned by *in silico* serotyping. The shade of the lines indicates nucleotide identity.

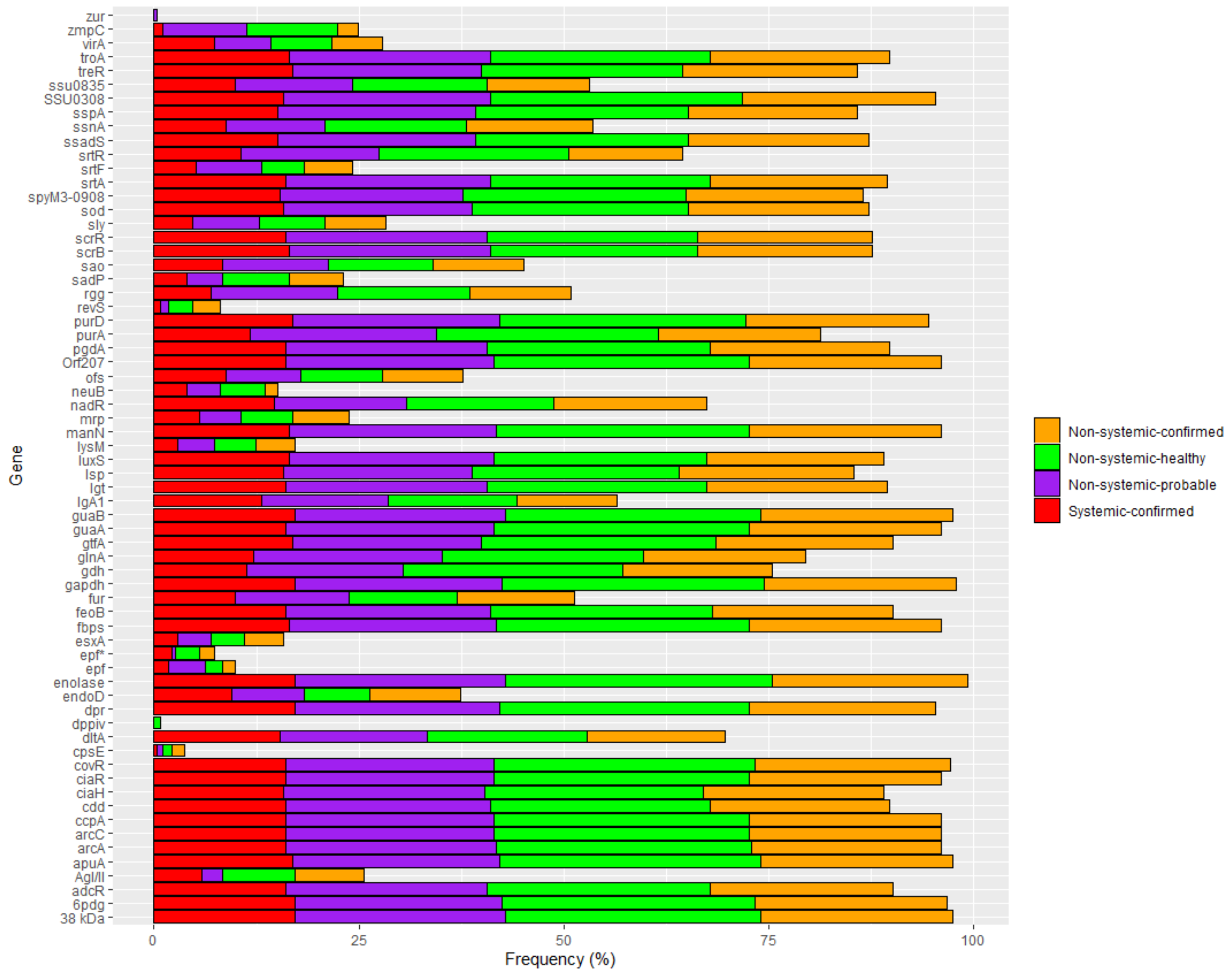


**S.D. Fig S2: Frequency (%) of all virulence associated factor genes detected in 273 *S. suis* isolates**

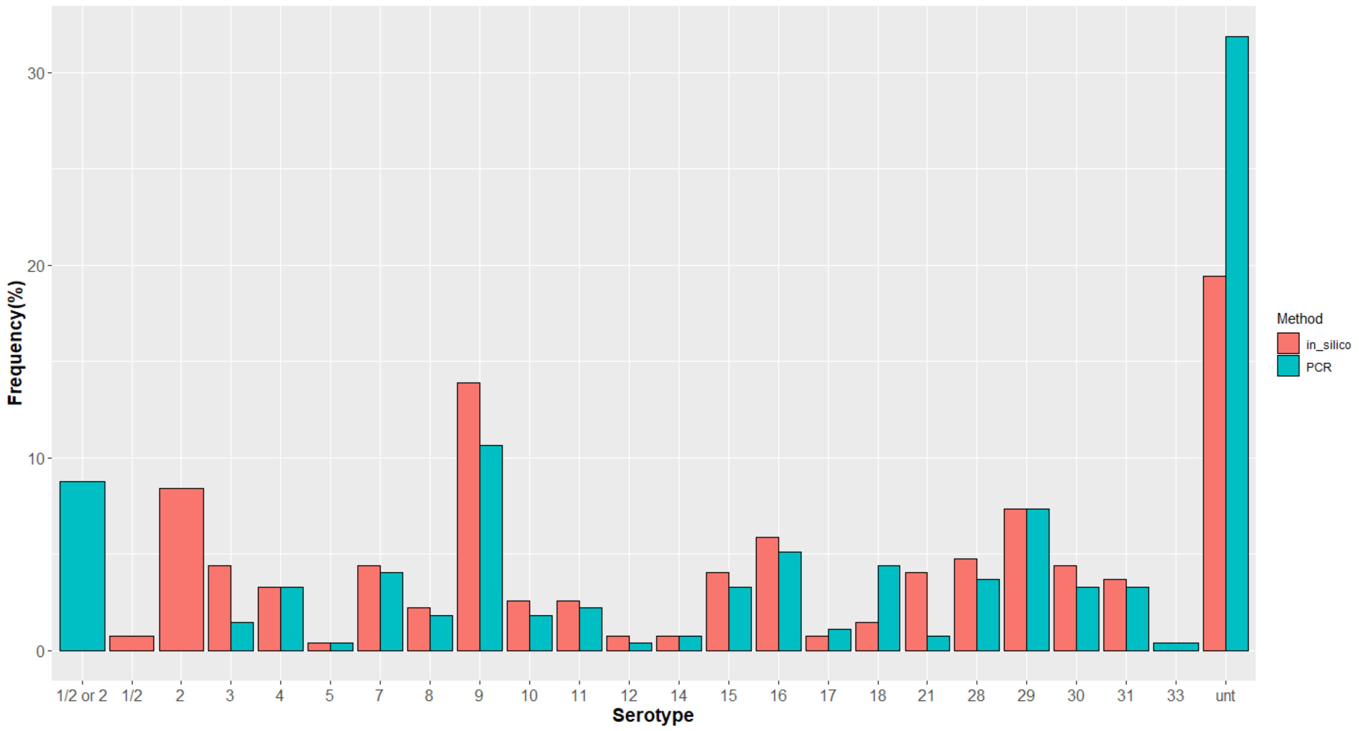


**S.D. Fig S3: Difference in serotypes identified between *in silico* and PCR serotyping methods (N=273)**

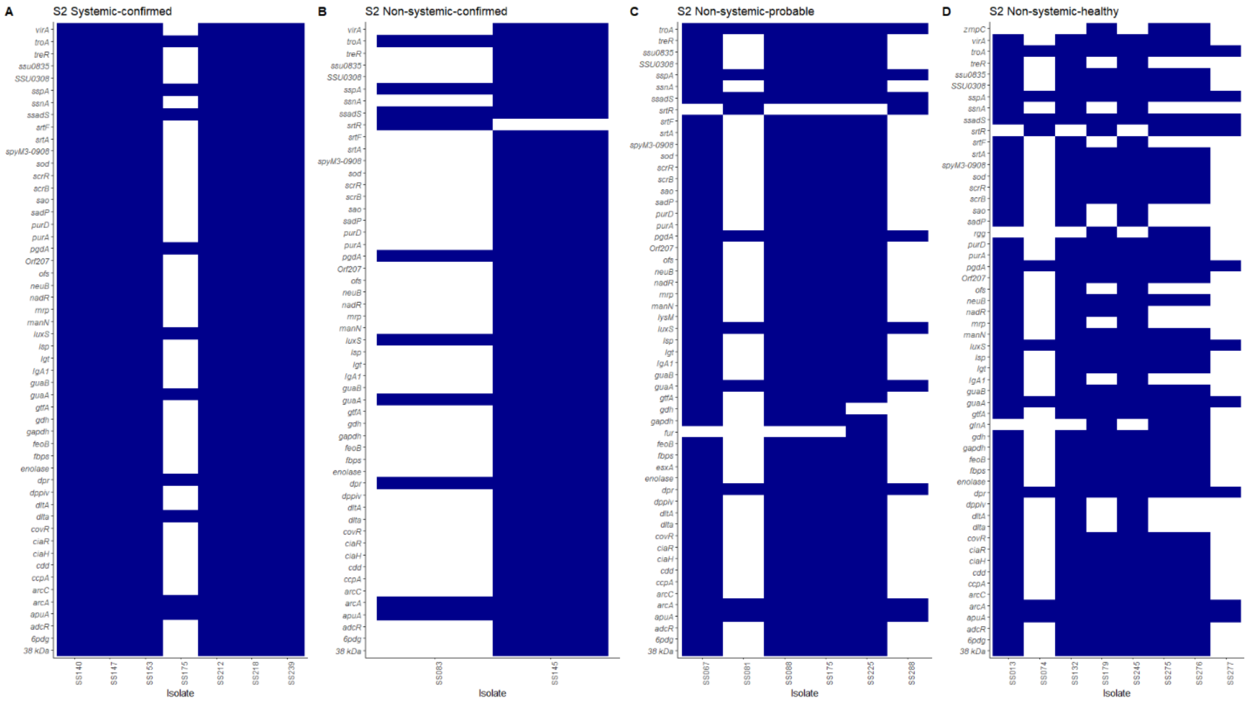


**S.D. Fig S4: Virulence associated factor profiles of 23 *S. suis* serotype 2 by SC, NSC, NSP and NSH isolate groups**

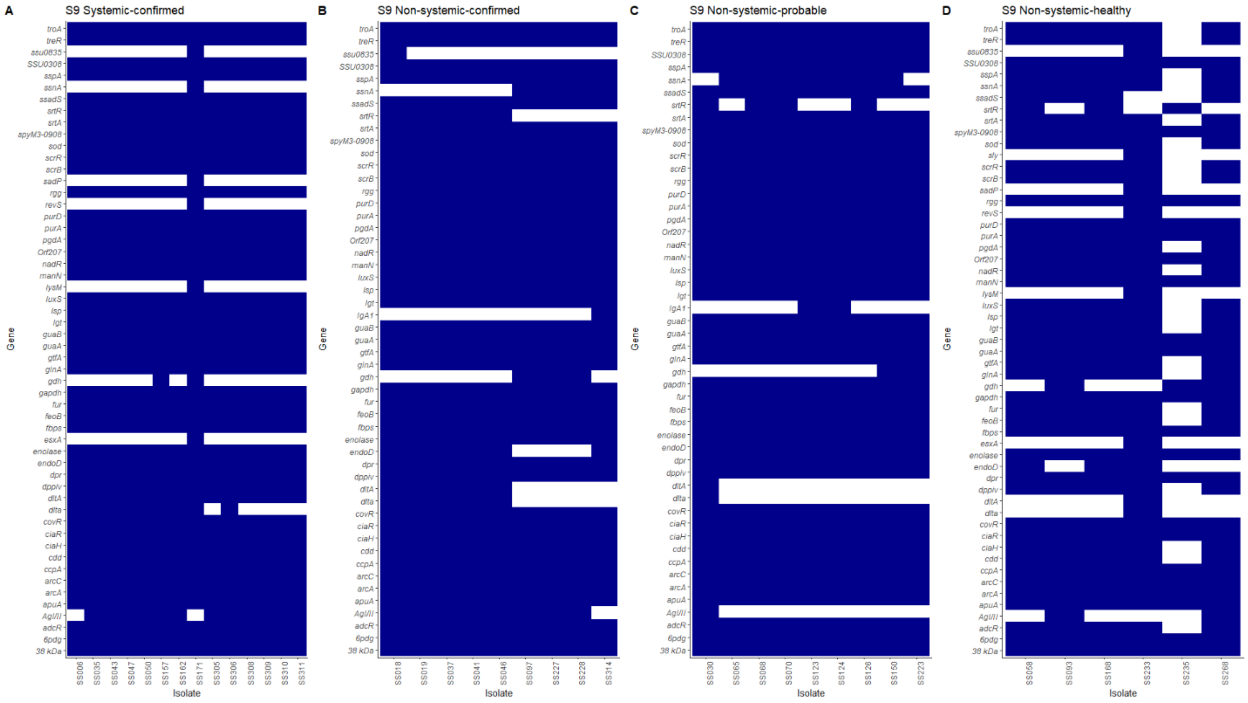


**S.D. Fig S5: VAF profiles of 38 *S. suis* serotype 9 by SC, NSC, NSP and NSH isolate groups**

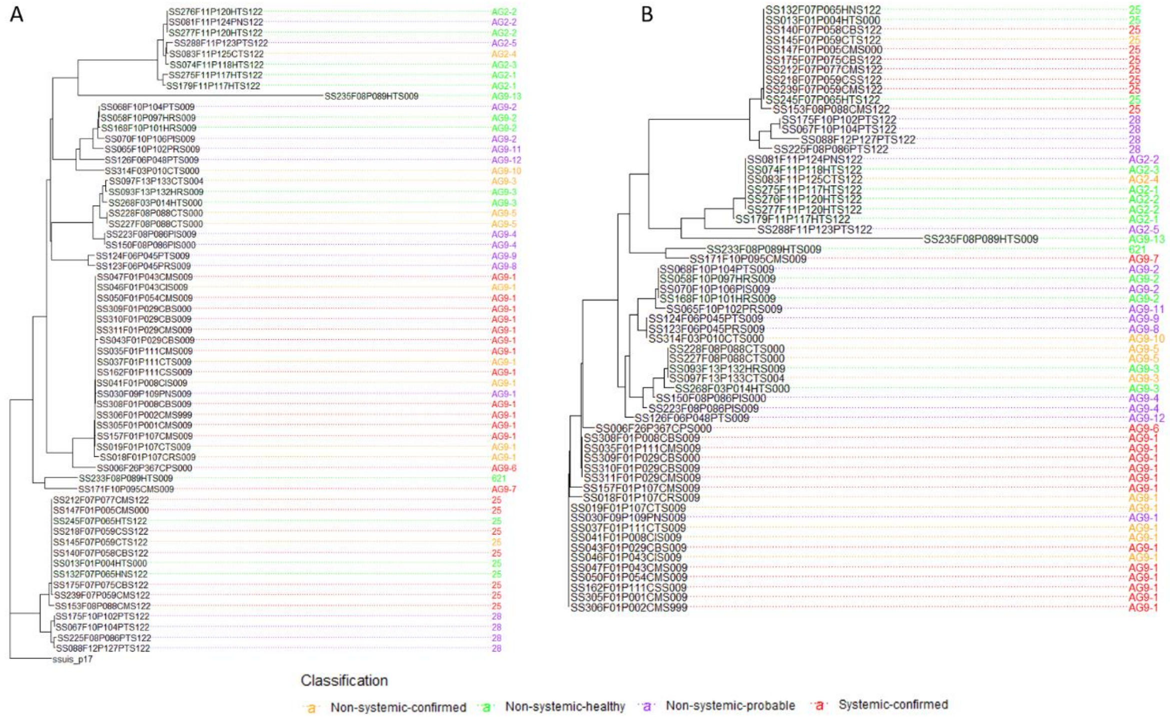


**S.D. Fig S6: Neighbour joining trees of serotype 2 and 9 isolates based on (A) MASH distances and (B) virulence associated factor presence and absence data**. Colored text denotes the sequence type (ST) if known and allele group if not known. The allele groups are based on the variants of the seven housekeeping genes used in MLST of *S. suis*: *aroA*, *cpn60*, *dpr*, *gki*, *mutS*, *recA*, and *thrA*.

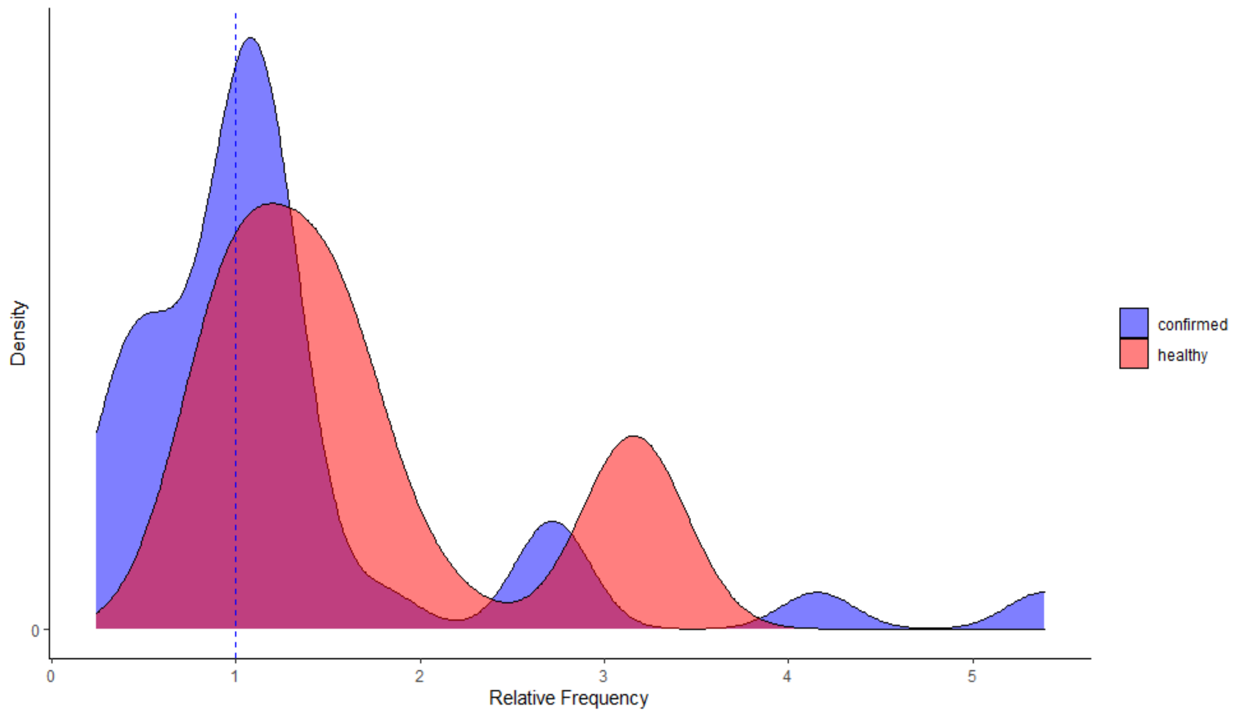


**S.D. Fig S7: Distribution of antimicrobial resistance genes in isolates from healthy pigs and pigs with confirmed *S. suis* infections.** Density plots of relative frequencies of AMR genes from healthy pigs (orange) and confirmed cases (blue). A relative frequency >1 denotes a higher prevalence of gene in a particular host pig health case.

## 1.2 Supplementary Tables

S.D. Tab S1: *Streptococcus suis* reference genomes

| Strain | Serotype | Accession |
|---|---|---|
| *Streptococcus suis* S735 | 2 | CP003736 |
| *Streptococcus suis* SC84 | 2 | NC_012924 |
| *Streptococcus suis* P1/7 | 2 | AM946016 |
| *Streptococcus suis* BM407 | 2 | NC_012926 |
| *Streptococcus suis* GZ1 | 2 | NC_017617 |
| *Streptococcus suis* JS14 | 2 | NC_017618 |
| *Streptococcus suis* 05HAS68 | 2 | NZ_CP002007 |
| *Streptococcus suis* NSUI060 | 2 | NZ_CP012911 |
| *Streptococcus suis* HN105 | 5 | NZ_CP029398 |
| *Streptococcus suis* D12 | 9 | CP002644 |
| *Streptococcus suis* DN13 | 9 | CP015557 |

S.D. Tab S2: Distribution of individual virulence associated factor (VAF) genes in clinical and non-clinical isolates.

| | Number (%) of isolates | | | Fisher's exact test (p-value) | | |
|---|---|---|---|---|---|---|
| VAF/genes | SC | NSC | NSH | SC vs NSC | SC vs NSH | NSC vs NSH |
| 38 kDa | 47(100) | 64(98) | 85(94) | 1.0000 | 0.1647 | 0.4018 |
| 6pdg | 47(100) | 64(98) | 84(93) | 1.0000 | 0.0939 | 0.2398 |
| adcR | 44(94) | 61(94) | 74(82) | 1.0000 | 0.0742 | 0.0501 |
| AgI/II | 16(34) | 23(35) | 24(27) | 1.000 | 0.7064 | 0.2891 |
| apuA | 46(97) | 64(98) | 87(97) | 1.000 | 0.6323 | 0.6398 |
| arcA | 44(94) | 63(97) | 85(94) | 0.5223 | 0.2572 | 0.4506 |
| arcC | 44(94) | 64(98) | 85(94) | 0.3074 | 1.0000 | 0.4018 |
| ccpA | 44(94) | 65(98) | 85(94) | 0.3074 | 1.0000 | 0.4018 |
| cdd | 44(94) | 60(92) | 73(81) | 1.0000 | 0.0725 | 0.0622 |
| ciaH | 43(91) | 60(92) | 73(81) | 1.0000 | 0.1372 | 0.0622 |
| ciaR | 44(94) | 64(98) | 85(94) | 0.3074 | 1.0000 | 0.4018 |
| covR | 44(94) | 65(100) | 87(97) | 0.0711 | 0.4126 | 0.2648 |
| dltA | 42(89) | 46(71) | 53(59) | 0.0204 | 0.0002 | 0.1750 |
| dpr | 47(100) | 62(95) | 83(92) | 0.2628 | 0.0952 | 0.5216 |
| enolase | 47(100) | 65(100) | 89(99) | 1.0000 | 1.0000 | 1.0000 |
| epf | 5(11) | 4(6) | 6(7) | 0.4881 | 0.5109 | 1.0000 |
| epf* | 6(13) | 5(8) | 8(9) | 0.5219 | 0.5556 | 1.0000 |
| fbps | 45(96) | 64(98) | 84(93) | 0.5711 | 0.7149 | 0.2398 |
| feoB | 44(94) | 60(92) | 74(82) | 1.0000 | 0.0742 | 0.0956 |
| gapdh | 47(100) | 64(98) | 87(97) | 1.0000 | 0.5510 | 0.6398 |
| gdh | 31(66) | 50(77) | 73(81) | 0.2087 | 0.0595 | 0.5513 |
| glnA | 33(70) | 54(83) | 67(74) | 0.1151 | 0.6859 | 0.2402 |
| guaA | 44(94) | 64(98) | 85(94) | 0.3070 | 1.0000 | 0.4018 |
| guaB | 47(100) | 64(98) | 85(94) | 1.0000 | 0.1647 | 0.4018 |
| lgt | 44(94) | 60(92) | 73(81) | 1.0000 | 0.0725 | 0.0622 |

| | | | | | | |
|---|---|---|---|---|---|---|
| luxS | 45(96) | 59(91) | 71(79) | 0.4644 | 0.0111 | 0.0503 |
| manN | 45(96) | 64(98) | 84(93) | 0.5711 | 0.7149 | 0.2398 |
| mrp | 15(32) | 19(29) | 17(19) | 0.8361 | 0.0941 | 0.1769 |
| pgdA | 44(94) | 60(92) | 74(82) | 0.5521 | 0.0742 | 0.0955 |
| purA | 32(68) | 54(83) | 74(82) | 0.0733 | 0.0845 | 1.0000 |
| purD | 46(98) | 61(94) | 82(91) | 0.3966 | 0.1645 | 0.7621 |
| rgg | 19(40) | 34(52) | 44(49) | 0.2522 | 0.3716 | 0.7455 |
| sly | 13(28) | 20(31) | 22(24) | 0.8344 | 0.6850 | 0.4643 |
| sod | 43(91) | 60(92) | 72(80) | 1.0000 | 0.0921 | 0.0398 |
| spyM3-0908 | 42(89) | 59(91) | 74(82) | 1.0000 | 0.3256 | 0.1645 |
| srtA | 44(94) | 59(91) | 73(81) | 0.7319 | 0.0725 | 0.1123 |
| ssadS | 41(87) | 60(92) | 71(79) | 0.5219 | 0.2551 | 0.0252 |
| ssnA | 41(51) | 42(65) | 47(52) | 0.1757 | 1.0000 | 0.1405 |
| sspA | 41(87) | 56(86) | 71(79) | 1.0000 | 0.2961 | 0.2834 |
| SSU0308 | 43(91) | 64(98) | 84(93) | 0.1594 | 0.7358 | 0.2398 |
| troA | 45(96) | 60(92) | 73(81) | 0.697 | 0.0193 | 0.06215 |

** SC = isolates from systemic sites in pigs with confirmed infections
** NSC = isolates from non-systemic sites in pigs with confirmed infections
** NSP = isolates from non-systemic sites in symptomatic pigs, which had no *S. suis* recovered from their systemic sites
** NSH = isolates from non-systemic sites in healthy pigs

S.D. Tab S3: Mixed effect logistic regression for gene presence in healthy and sick pigs with farm and serotype as random variables.

| Gene | Parameters | | Odds Ratio | Std. error | 95% CI | P- value |
|---|---|---|---|---|---|---|
| *dltA* | Fixed effects: Isolate source | Non-systemic-healthy | Reference | | | |
| | | Non-systemic-confirmed | 1.183 | 0.064 | 0.984, 1.265 | 0.090 |
| | | Non-systemic-probable | 1.808 | 0.063 | 1.046, 1.337 | 0.010 |
| | | Systemic-confirmed | 1.116 | 0.072 | 1.140, 1.513 | < 0.001 |
| | Random effects | Serotype | -- | 0.286 | -- | < 0.001 |
| | | Farm | -- | 0.134 | -- | < 0.001 |
| | | | | | | |
| *luxS* | Fixed effects: Isolate source | Non-systemic-healthy | Reference | | | |
| | | Non-systemic-confirmed | 1.184 | 0.046 | 0.994, 1.190 | 0.069 |
| | | Non-systemic-probable | 2.279 | 0.044 | 1.087, 1.290 | < 0.001 |
| | | Systemic-confirmed | 1.088 | 0.052 | 0.998, 1.224 | 0.069 |
| | Random effects | Serotype | -- | 0.149 | -- | < 0.001 |
| | | Farm | -- | 0.000 | -- | 1.000 |
| | | | | | | |
| *troA* | Fixed effects: Isolate source | Non-systemic-healthy | Reference | | | |
| | | Non-systemic-confirmed | 1.135 | 0.045 | 0.994, 1.184 | 0.077 |
| | | Non-systemic-probable | 2.303 | 0.042 | 1.044, 1.232 | 0.006 |
| | | Systemic-confirmed | 1.085 | 0.050 | 0.991, 1.207 | 0.077 |
| | Random effects | Serotype | -- | 0.162 | -- | < 0.001 |
| | | Farm | -- | 0.000 | -- | 1.000 |

“--" = not applicable

S.D. Tab S4: Mixed effect logistic regression for distinct genes in serotype 2 and 9 SC isolates.

| Gene | Parameters | | Odds Ratio | Std. error | 95% CI | P- value |
|---|---|---|---|---|---|---|
| *AgI/II* | Fixed effects: Isolate source | Non-systemic-healthy | Reference | | | |
| | | Non-systemic-confirmed | 1.029 | 0.029 | 0.988, 1.107 | 0.165 |
| | | Non-systemic-probable | 2.574 | 0.028 | 0.974, 1.087 | 0.300 |
| | | Systemic-confirmed | 1.046 | 0.032 | 0.989, 1.122 | 0.165 |
| | Random effects | Serotype | -- | 0.070 | -- | 0.004 |
| | | Farm | -- | 0.029 | -- | 0.088 |
| | | | | | | |
| *dpr* | Fixed effects: Isolate source | Non-systemic-healthy | Reference | | | |
| | | Non-systemic-confirmed | 1.020 | 0.035 | 0.968, 1.111 | 0.384 |
| | | Non-systemic-probable | 2.561 | 0.034 | 0.955, 1.091 | 0.551 |
| | | Systemic-confirmed | 1.038 | 0.039 | 0.988, 1.151 | 0.195 |
| | Random effects | Serotype | -- | 0.064 | -- | 0.002 |
| | | Farm | -- | 0.043 | -- | 0.042 |
| | | | | | | |
| *fur* | Fixed effects: Isolate source | Non-systemic-healthy | Reference | | | |
| | | Non-systemic-confirmed | 1.017 | 0.061 | 1.031, 1.308 | 0.030 |
| | | Non-systemic-probable | 1.535 | 0.059 | 0.907, 1.141 | 0.775 |
| | | Systemic-confirmed | 1.161 | 0.069 | 0.939, 1.230 | 0.399 |
| | Random effects | Serotype | -- | 0.371 | -- | 0.000 |
| | | Farm | -- | 0.073 | -- | 0.080 |
| | | | | | | |
| *guaA* | Fixed effects: Isolate source | Non-systemic-healthy | Reference | | | |
| | | Non-systemic-confirmed | 1.024 | 0.032 | 0.968, 1.096 | 0.428 |
| | | Non-systemic-probable | 2.585 | 0.030 | 0.965, 1.086 | 0.428 |
| | | Systemic-confirmed | 1.030 | 0.035 | 0.900, 1.035 | 0.428 |
| | Random effects | Serotype | -- | 0.081 | -- | 0.001 |
| | | Farm | -- | 0.015 | -- | 0.726 |
| | | | | | | |
| *mrp* | Fixed effects: | Non-systemic-healthy | Reference | | | |

| | | | | | | |
|---|---|---|---|---|---|---|
| | Isolate source | Non-systemic-confirmed | 0.990 | 0.053 | 1.002, 1.231 | 0.094 |
| | | Non-systemic-probable | 1.230 | 0.051 | 0.895, 1.095 | 0.845 |
| | | Systemic-confirmed | 1.111 | 0.059 | 0.985, 1.239 | 0.123 |
| | Random effects | Serotype | -- | 0.302 | -- | < 0.001 |
| | | Farm | -- | 0.111 | -- | < 0.001 |
| | | | | | | |
| *ofs* | Fixed effects: Isolate source | Non-systemic-healthy | Reference | | | |
| | | Non-systemic-confirmed | 1.106 | 0.052 | 0.967, 1.187 | 0.187 |
| | | Non-systemic-probable | 1.520 | 0.050 | 1.002, 1.220 | 0.062 |
| | | Systemic-confirmed | 1.072 | 0.059 | 1.028, 1.295 | 0.033 |
| | Random effects | Serotype | -- | 0.385 | -- | < 0.001 |
| | | Farm | -- | 0.080 | -- | 0.007 |
| | | | | | | |
| *pgdA* | Fixed effects: Isolate source | Non-systemic-healthy | Reference | | | |
| | | Non-systemic-confirmed | 1.119 | 0.045 | 0.982, 1.172 | 0.157 |
| | | Non-systemic-probable | 2.335 | 0.043 | 1.028, 1.217 | 0.019 |
| | | Systemic-confirmed | 1.073 | 0.051 | 0.955, 1.165 | 0.301 |
| | Random effects | Serotype | -- | 0.161 | -- | < 0.001 |
| | | Farm | -- | 0.022 | -- | 0.701 |
| | | | | | | |
| *srtF* | Fixed effects: Isolate source | Non-systemic-healthy | Reference | | | |
| | | Non-systemic-confirmed | 1.124 | 0.051 | 1.035, 1.266 | 0.015 |
| | | Non-systemic-probable | 1.227 | 0.049 | 1.021, 1.238 | 0.020 |
| | | Systemic-confirmed | 1.146 | 0.057 | 1.066, 1.331 | 0.009 |
| | Random effects | Serotype | -- | 0.330 | -- | < 0.001 |
| | | Farm | -- | 0.082 | -- | 0.021 |
| | | | | | | |
| *virA* | Fixed effects: Isolate source | Non-systemic-healthy | Reference | | | |
| | | Non-systemic-confirmed | 1.129 | 0.044 | 0.939, 1.116 | 0.601 |
| | | Non-systemic-probable | 1.357 | 0.043 | 1.038, 1.229 | 0.010 |

| | | | | | | |
|---|---|---|---|---|---|---|
| | | Systemic-confirmed | 1.023 | 0.050 | 0.960, 1.167 | 0.349 |
| | Random effects | Serotype | -- | 0.402 | -- | < 0.001 |
| | | Farm | -- | 0.096 | -- | 0.004 |

“--" = not applicable

S.D. Tab S5: Carriage distribution of antimicrobial resistance genes in *S. suis* isolates.

| | Number (%) of isolates | | | | |
|---|---|---|---|---|---|
| Number of AMR-associated genes carried | SC | NSC | NSH | NSP | All Isolates |
| 1 | 0(0) | (0) | 1(1) | 1(1) | 2(1) |
| 2 | 30(64) | 34(46) | 34(38) | 34(48) | 128(47) |
| 3 | 10(21) | 14(22) | 17(19) | 10(14) | 51(19) |
| 4 | 3(6) | 7(11) | 10(11) | 8(11) | 28(10) |
| 5 | 3(6) | 7(11) | 9(10) | 7(10) | 26(10) |
| 6 | 1(2) | 4(6) | 9(10) | 7(10) | 21(8) |
| 7 | 0(0) | 1(2) | 5(6) | 1(1) | 7(3) |
| 8 | 0(0) | 0(0) | 2(2) | 2(3) | 4(1) |

** SC = isolates from systemic sites in pigs with confirmed infections
** NSC = isolates from non-systemic sites in pigs with confirmed infections
** NSP = isolates from non-systemic sites in symptomatic pigs, which had no *S. suis* recovered from their systemic sites
** NSH = isolates from non-systemic sites in healthy pigs

S.D. Tab S6: Carriage distribution of potential antimicrobial resistance to different drug classes in *S. suis* isolates.

| | Number(%) of isolates | | | | |
|---|---|---|---|---|---|
| Number of drug class carried | SC | NSC | NSH | NSP | All Isolates |
| 1 | 0(0) | 0(0) | 3(3) | 1(1) | 4(1) |
| 2 | 0(0) | 2(3) | 4(4) | 1(1) | 7(3) |
| 3 | 1(2) | 5(8) | 2(2) | 1(1) | 9(3) |
| 4 | 33(70) | 28(43) | 39(43) | 40(56) | 140(51) |
| 5 | 8(17) | 10(15) | 14(16) | 8(11) | 10(15) |
| 6 | 1(2) | 0(0) | 5(6) | 6(8) | 12(4) |
| 7 | 3(6) | 10(15) | 9(10) | 3(4) | 25(9) |
| 8 | 0(0) | 5(8) | 7(8) | 7(10) | 19(7) |
| 9 | 0(0) | 3(5) | 3(3) | 3(4) | 9(3) |
| 10 | 1(2) | 0(0) | 1(1) | 0(0) | 2(1) |

** SC = isolates from systemic sites in pigs with confirmed infections
** NSC = isolates from non-systemic sites in pigs with confirmed infections
** NSP = isolates from non-systemic sites in symptomatic pigs, which had no *S. suis* recovered from their systemic sites
** NSH = isolates from non-systemic sites in healthy pigs
